# Next-generation protocol design for blockchain rewards with web-tool

**DOI:** 10.1038/s41598-025-28161-9

**Published:** 2025-11-27

**Authors:** Muhammad Zeshan Arshad, Ali Algarni

**Affiliations:** 1 Department of Mathematics and Statistics, University of Agriculture, Faisalabad, Pakistan; 2 Faculty of science, Department of Statistics, King Abdulaziz University, Jeddah, Saudi Arabia

**Keywords:** Blockchain, Mining reward, Miner heterogeneity, Entropy measure, Simulation, Shiny dashboard, Engineering, Mathematics and computing, Physics

## Abstract

Designing fair and efficient blockchain reward mechanisms requires going beyond raw execution time to account for behavioral variability. We present a simulation framework for evaluating BCRPs using entropy as a systems-level indicator of reward fairness and stability. Three strategies are assessed on simulated miner profiles $\:(n=100)$ with log-normal execution times, Laplace-distributed noise, and tercile-based complexity classes: a classical execution-time baseline, “Mining $\:2.0$” (penalizing miner noise and task complexity), and “Adaptive $\:2.0$” (Mining $\:2.0$ with exponential time decay). Reward distributions are summarized via KDE and ECDF and scored using Shannon, Rényi $\:(\alpha\:=2)$, Tsallis $\:(q=2)$, and normalized Shannon entropies computed on discretized rewards ($\:20$ bins). An interactive Shiny application accompanies the method for reproducible exploration without programming. Across simulations, Adaptive $\:2.0$ yields the most behavior-sensitive and equitable allocations, achieving the lowest entropy on all four metrics. Quantitatively, relative to the Traditional baseline, Adaptive $\:2.0$ reduces entropy by $\:37.5\%$ (Shannon: $\:2.684\to\:1.678$), $\:37.1\%$ (Rényi-$\:2$: $\:2.218\to\:1.396$), $\:21.0\%$ (Tsallis-$\:2$: $\:0.785\to\:0.620$), and $\:14.6\%$ (Normalized: $\:0.847\to\:0.723$); Mining $\:2.0$ achieves intermediate improvements of $\:29.8\%,\:31.7\%,\:17.2\%,$ and $\:13.9\%,$ respectively. These results provide an evidence-based, deployable framework for evaluating reward fairness in decentralized systems.

## Introduction

From financial systems to hospital networks to supply chains, blockchain technology has evolved into a flexible platform for distributed applications allowing safe, transparent, and tamper-resistant data transmission. Every blockchain system is fundamentally founded on an incentive mechanism—a rule-based system controlling how players are paid for preserving network integrity via consensus involvement. Ensuring miner involvement, system sustainability, and resilience to adversary manipulation depends critically on the fairness and efficiency of these incentive systems.

Anagnostakis and Glavas^1^ showed that adding more witnesses lowers entropy and increases system stability, hence offering a new measure based on Shannon entropy and Lyapunov dynamics. Using entropy for data compression and effective block validation, Vopson et al.^2^ presented the Entropic Blockchain. Likewise, Liu et al.^3^ established entropy as a strong instrument for assessing structural complexity and behavioral sensitivity in blockchain-based economies by using Kolmogorov entropy to investigate market chaos in cryptocurrency systems and smart cities. Beyond broad assessments, entropy has also been used in incentive design. Chen et al.^4^ using entropy as a surrogate for vote disagreement created an entropy-driven payment system to fight false information in distributed networks. To rank blockchain systems by sustainability and performance. Zolfaghari et al.^5^ underlined once again the fundamental role entropy plays in cryptographic security. Todorović et al.^6^ and Singh et al.^7^ investigated reward dynamics using optimization and game-theoretic techniques, respectively. While Zafar et al.^8^ coupled entropy and CRITIC-based weighting with MCDM approaches. Introduced by Nakamoto^9^, the traditional reward system pays miners depending only on computing work and execution speed. Although good in early peer-to-peer bitcoin systems like Bitcoin, this approach has major restrictions. It ignores important considerations such behavioral noise, operational complexity, and systematic volatility and assumes homogeneous miner behavior.

In parallel, the IoT/AI security literature has advanced rapidly. Federated learning is increasingly used to support privacy-preserving, bandwidth-aware learning at the edge^10^; blockchain–IoT integration has been surveyed as a means to provide integrity, auditability, and trust for constrained devices^11^; generative-AI/ML defenses for IoT security are synthesized in recent reviews^12^; post-quantum security considerations for blockchain-IoT deployments are mapped in detail^13^; and AI-based intrusion detection for cyber-physical/vehicular networks illustrates deployment-level detection pipelines^14^. These strands position our contribution as complementary—an entropy-based evaluation of incentive fairness that can be layered alongside AI-enabled IoT security stacks.

Notwithstanding these developments, a clear research vacuum remains: no current work operationalizes entropy as a measure for assessing reward fairness across blockchain protocols in the context of miner heterogeneity. Particularly lacking are models that replicate real-world behavioral diversity—such as computational complexity and execution instability—while quantitatively evaluating their influence on reward dispersion and fairness using entropy-based criteria.

Focusing on miner heterogeneity and entropy-driven fairness analysis, this paper closes that gap by offering a simulation-based assessment approach for blockchain reward systems. We offer two improved models based on the Traditional methodology as a baseline:

- Mining 2.0, which incorporates miner noise and task complexity into reward allocation, and.
- Adaptive 2.0, which further applies exponential decay to penalize inefficient execution times, modeling temporal degradation in miner performance.

To evaluate these protocols, we simulate miner populations characterized by log-normal execution times, Laplacian-distributed behavioral noise, and stratified complexity classes. Reward distributions are analyzed using KDE, ECDFs, and four entropy measures—Shannon, Rényi, Tsallis, and Normalized Entropy—to capture variability, unpredictability, and fairness.

A unique feature of this work is the development of an interactive Shiny-based web-tool that encapsulates the full simulation and analytical framework. This tool allows users to dynamically explore miner performance, reward sensitivity, and entropy outcomes without requiring programming expertise—bridging the gap between theoretical modeling and practical usability.

In doing so, this study provides an accessible, extensible, and statistically grounded approach to reward mechanism evaluation. It contributes not only two novel reward protocols but also a deployable research tool that can aid future blockchain designers in developing behavior-aware, entropy-informed, and fair incentive structures.

### Study theme and approach

This study introduces a simulation-driven framework for designing and evaluating next-generation blockchain reward protocols (BCRPs). Existing mechanisms—represented by the Traditional protocol (baseline)—typically allocate rewards based solely on execution time or computational effort, overlooking the behavioral and operational variability inherent in decentralized systems. To address these limitations, we simulate a synthetic miner population characterized by log-normal execution times, Laplacian-distributed behavioral noise, and stratified task complexity (low, medium, high).

Building on the baseline, we propose and analyze two refined protocols: Mining 2.0 (Protocol 2.0), which adjusts rewards based on miner noise and task complexity, and Adaptive 2.0, which further incorporates execution-time sensitivity by applying exponential decay to penalize delays and model diminishing efficiency. These strategies reflect progressively behavior-aware reward designs that account for miner heterogeneity and protocol responsiveness.

To assess the structure and fairness of the resulting reward distributions, we employ kernel density estimation (KDE) and empirical cumulative distribution functions (ECDFs). A key novelty of this study is the integration of multiple entropy-based measures—Shannon, Rényi, Tsallis, and Normalized Entropy—to quantify randomness, dispersion, and sensitivity across protocols. These metrics offer a statistically grounded and interpretable framework for evaluating protocol performance.

A significant contribution to this work is the development of an interactive Shiny-based web-tool that operationalizes the simulation architecture for broader usability. Without requiring programming expertise, users can simulate miner populations, visualize reward outcomes, and compare protocol dynamics. Together, the modeling framework and web interface provide a transparent, extensible, and behavior-sensitive foundation for evaluating blockchain incentive mechanisms, supporting the design of fairer, more robust decentralized systems.

This work includes the following contributions:

A simulation framework that evaluates blockchain reward protocols using entropy-based fairness/stability metrics on discretized rewards ($\:20\:$bins).

- Two behavior-aware reward designs Mining $\:2.0$ (noise- and complexity-aware) and Adaptive $\:2.0$ (adds exponential time decay).
- A deployable Shiny tool for reproducible, no-code exploration of miner behavior and reward outcomes.
- Quantitative evidence that Adaptive $\:2.0$ achieves the lowest entropy across all metrics versus the Traditional baseline: Shannon $\:2.684\to\:1.678\:(-37.5\%)$, Rényi-2 $\:2.218\to\:1.396\:(-37.1\%),\:$Tsallis-2 $\:0.785\to\:0.620\:(-21.0\%),$ Normalized $\:0.847\to\:0.723\:(-14.6\%).$ Mining $\:2.0$ yields intermediate gains: Shannon $\:2.684\to\:1.885\:(-29.8\%),$ Rényi-2 $\:2.218\to\:1.515\:(-31.7\%),$ Tsallis-2 $\:0.785\to\:0.650\:(-17.2\%),$ Normalized $\:0.847\to\:0.729\:(-13.9\%)$ (Table 1).

**Table 1:** Entropy measures for reward distributions across protocols.

Protocol	Shannon	Renyi	Tsallis	Normalized
Traditional	2.68418372	2.217591435	0.785	0.846765686
Mining 2.0	1.885475297	1.514573173	0.65	0.729401412
Adaptive 2.0	1.678389825	1.395928676	0.62	0.722843153

 The remainder of this paper is organized as follows: Sect. 2 details the simulation setup and entropy measures; Sect. 3 formalizes protocol evaluation; Sect. 4 reports results and Sect. 5 makes comprehensive discussion; Sect. 6 presents conclusions and Sect. 7 present the future work and limits.

## Methodology

This study simulates miner behavior using log-normal execution times, Laplace-distributed noise, and complexity-based classification. Three reward protocols are modeled: the Traditional protocol (baseline), and two proposed protocols—Protocol 2.0, which incorporates noise and complexity, and Adaptive 2.0, which adds an exponential penalty for delayed execution. Reward outcomes are evaluated using KDE, ECDFs, and four entropy measures—Shannon, Rényi, Tsallis, and Normalized Entropy—to quantify fairness and structural variation. Additional diagnostics, such as noise-effort tradeoffs and class-based comparisons, further assess protocol behavior. All simulations and analyses are operationalized through a Shiny-based web-tool, allowing users to explore results interactively via a code-free, user-friendly interface.

### Miner simulation

To simulate a realistic blockchain environment, we generate a population of one hundred (*n* = 100) synthetic miners. Each miner is defined by three core attributes that reflect behavioral and computational diversity: execution time, miner noise, and task complexity.

#### Execution time ($\:{\left.T\right|}_{j}$)

The execution times are simulated using a log-normal distribution, which captures the right-skewed processing delays commonly observed in real-world computing systems:

1 $$\:{\left.T\right|}_{j}\sim\mathrm{L}\mathrm{o}\mathrm{g}\mathrm{N}\mathrm{o}\mathrm{r}\mathrm{m}\mathrm{a}\mathrm{l}\left(\mu\:=1.5,\:\sigma\:=0.5\right).$$

Note that this distribution allows for a majority of miners to complete tasks within a moderate range while also accommodating high-latency outliers.

#### Miner noise ($\:{\left.N\right|}_{j}$)

The behavioral noise is introduced using a Laplace distribution centered at 1. This models erratic performance or external disturbances in signal processing:

2 $$\:{\left.N\right|}_{j}\sim\mathrm{L}\mathrm{a}\mathrm{p}\mathrm{l}\mathrm{a}\mathrm{c}\mathrm{e}\left(\mu\:=1,\:\sigma\:=0.5\right)\:$$

Essentially, the Laplace distribution’s heavier tails accommodate irregular miner behavior, including burstiness and instability. Note that $\:\mu\:$ (mean) and $\:\sigma\:$ (standard deviation) of Laplace distribution, respectively.

#### Complexity class ($\:{\left.C\right|}_{j}$)

Complexity scores are assigned by ranking all miners based on their execution time and dividing them into three equal terciles:

3 $$\:{\left.C\right|}_{j}=\left\{\begin{array}{c}1,\:\:\:\:\:\:\:\:\:\:\:\:\:\:\:\:\:\:\:\:\:\:\:\:\:if\:{\left.T\right|}_{j}\le\:{\left.Q\right|}_{1/3},\:\\\:2,\:\:if\:{\left.Q\right|}_{1/3}<{\left.T\right|}_{j}\le\:{\left.Q\right|}_{2/3},\\\:3,\:\:\:\:\:\:\:\:\:\:\:\:\:\:\:\:\:\:\:\:\:\:\:\:\:\:\:\:\:\:\:{\left.T\right|}_{j}>{\left.Q\right|}_{2/3}.\end{array}\right.$$

Note that $\:{\left.Q\right|}_{1/3}$and $\:{\left.Q\right|}_{2/3}$represent the 33rd and 66th percentiles of distribution. This classification acts as a proxy for computational burden, where higher complexity indicates more time-intensive tasks. This simulation framework provides a diverse and behaviorally rich miner profile to test protocol responses under varying conditions.

### Protocol formulations

We define three reward-generation strategies that represent progressively more sophisticated views of miner performance and system responsiveness.

#### Traditional protocol (baseline)

As first proposed by Nakamoto^11^, the Traditional protocol rewards miners purely based on how fast they complete a task—faster execution times yield higher rewards:

4 $$\:{\left.R\right|}_{j}^{\left(\mathrm{T}\mathrm{r}\mathrm{a}\mathrm{d}\mathrm{i}\mathrm{t}\mathrm{i}\mathrm{o}\mathrm{n}\mathrm{a}\mathrm{l}\right)}=\frac{1}{{\left.T\right|}_{j}}\times\:\frac{10}{{\left.\mathrm{M}\mathrm{a}\mathrm{x}\right|}_{T}}$$

Assuming quicker miners are always more meritorious, this algorithm normalizes the reward to a 0–10 scale disregarding work difficulty or noise.

#### Protocol 2.0

The proposed protocol 2.0 introduces a more sophisticated approach that discourages unpredictable or too resource-intensive activity by including miner noise and complexity class:

5 $$\:{\left.R\right|}_{j}^{\left(2.0\right)}=\frac{1}{{\left.C\right|}_{j}\times\:{\left.N\right|}_{j}}$$

It is important to recognize that rewards are subject to penalties due to instability, which can arise from a higher $\:{\left.N\right|}_{j}$ or increased computational demand represented by a higher $\:{\left.C\right|}_{j}$. This signifies a more sophisticated reward system that promotes equilibrium and effectiveness.

#### Adaptive protocol

The adaptive protocol builds Protocol 2.0 by adding an exponential decay mechanism imposing extra penalties for extended running times:

6 $$\:{\left.R\right|}_{j}^{\left(\mathrm{A}\mathrm{d}\mathrm{a}\mathrm{p}\mathrm{t}\mathrm{i}\mathrm{v}\mathrm{e}\right)}=\frac{1}{{\left.C\right|}_{j}\times\:{\left.N\right|}_{j}}\times\:{e}^{-\lambda\:{\left.T\right|}_{j}},\:\:\:\lambda\:=0.1$$

The decay constant is tuned to strike a mix between sensitivity and reward inhibition. Even for generally low-complexity, stable miners, this paradigm introduces decreasing returns over longer runtimes.

#### Reward distribution analysis

Statistical visualization techniques are employed to analyze the reward distributions in order to assess fairness and variability across protocols. The distributional equity across miners is visualized by constructing kernel density estimates and empirical cumulative distribution functions (ECDFs) for each protocol—Traditional, 2.0, and Adaptive.

Let the reward vector under protocol $\:k \in${Traditional, 2.0, Adaptive} be:

$$\:{\left.R\right|}_{n}^{\left(k\right)}=\left\{{\left.R\right|}_{1}^{\left(k\right)},{\left.R\right|}_{1}^{\left(k\right)},{\left.R\right|}_{1}^{\left(k\right)},\dots\:,\:{\left.R\right|}_{n}^{\left(k\right)}\right\},$$

Then the estimated reward density function $\:{\left.f\right|}_{r}^{\left(k\right)}$ is obtained using a Gaussian kernel:

$$\:{\left.f\right|}_{r}^{\left(k\right)}=\frac{1}{hn}\sum\:_{m=1}^{n}K\left(\frac{r-{\left.R\right|}_{m}^{\left(k\right)}}{h}\right),$$

$$\:{\left.K\right|}_{u}=\frac{1}{\surd\:2\pi\:}{e}^{-{u}^{2}/2},$$

Hence, the ECDF is defined as:

7 $$\:{\left.F\right|}_{r}^{\left(k\right)}=\frac{1}{n}\sum\:_{m=1}^{n}1\left\{{\left.R\right|}_{m}^{\left(k\right)}\le\:r\right\}.$$

These functions allow us to visually compare reward equity and spread across all protocols. Furthermore, we stratified miners by *complexity class* $\:{\left.C\right|}_{j} \in${1,2,3} and examined reward distributions within each class using boxplots and grouped bar charts. Let:

8 $$\:{\left.\mu\:\right|}_{j}^{\left(k\right)}=\frac{1}{{\left.n\right|}_{d}}\sum\:_{m \in {\left.D\right|}_{d}}{\left.R\right|}_{m}^{\left(k\right)},$$

where $\:{\left.D\right|}_{d}=\left\{j:{\left.D\right|}_{d}=c\right\}.$ Not that, this enables class-wise fairness comparison across protocols.

#### Miner behavior diagnostics

To further understand miner heterogeneity, we analyzed the relationship between miner instability $\:{\left.N\right|}_{j}$ and execution time $\:{\left.T\right|}_{j}$. These behavioral traits reflect underlying effort, efficiency, and environmental stability of miners, which affect reward outcomes under different protocols. We examined the joint distribution of $\:{\left.N\right|}_{j}$ and $\:{\left.T\right|}_{j}$using scatter plots and density functions.

Let the joint miner behavior space be:

9 $$\:B=\left\{\left({\left.N\right|}_{j},\:{\left.T\right|}_{j}\right):\:j=\mathrm{1,2},3,\dots\:,n\right\}$$

We visually assessed this using:

- Scatter plots: $$\:\left({\left.N\right|}_{j},\:{\left.T\right|}_{j}\right)$$
- Density estimates of $\:{\left.N\right|}_{j}$ and $\:{\left.T\right|}_{j}$ across complexity classes $\:{\left.C\right|}_{j}$.

These diagnostics highlight clusters, outliers, and trade-offs between signal instability and computational burden, offering insights into miner typologies. Additionally, we explored the dependency of rewards on instability by plotting $\:{\left.R\right|}_{m}^{\left(k\right)}$ against $\:{\left.N\right|}_{j}$, which reveals protocol sensitivity to noisy or erratic miners.

Moreover, KDE is applied to visualize the marginal distributions of both execution time and miner noise across complexity classes, highlighting skewness, concentration, and distributional spread.

### Entropy

To go beyond average rewards, we employ entropy measures that quantify the variability, unpredictability, and fairness of each protocol’s reward distribution. We discretize each reward vector into 20 bins and compute four types of entropy for each protocol:

#### Shannon entropy

This captures the average information content or randomness in reward allocation. A higher value indicates more equitable or diverse distributions.

10 $$\:{\left.H\right|}_{k}^{\left(\mathrm{S}\mathrm{h}\mathrm{a}\mathrm{n}\mathrm{n}\mathrm{o}\mathrm{n}\right)}=-\sum\:{\left.r\right|}_{k}lo{\mathrm{g}}_{2}{\left.r\right|}_{k}$$

#### Rényi entropy

Unlike Shannon, Rényi entropy gives more weight to dominant probabilities, making it suitable for detecting reward concentration or inequality.

11 $$\left. H \right|_{2}^{{\left( {Renyi} \right)}} = \frac{1}{{1 - 2}}\log _{2} \sum {r|_{k}^{2} }$$

#### Tsallis entropy

This generalization of entropy is widely used in non-extensive systems and is ideal for systems where, entropy scales non-linearly.

12 $$\:{\left.H\right|}_{2}^{\left(\mathrm{T}\mathrm{s}\mathrm{a}\mathrm{l}\mathrm{l}\mathrm{i}\mathrm{s}\right)}=\frac{1-\sum\:{\left.r\right|}_{k}^{2}}{1}$$

#### Normalized Shannon entropy

This ratio of observed to maximum entropy gives a normalized fairness score between 0 and 1.

13 $$\:{\left.H\right|}_{\mathrm{N}\mathrm{o}\mathrm{r}\mathrm{m}\mathrm{a}\mathrm{l}\mathrm{i}\mathrm{z}\mathrm{e}\mathrm{d}}^{\left(\mathrm{S}\mathrm{h}\mathrm{a}\mathrm{n}\mathrm{n}\mathrm{o}\mathrm{n}\right)}=\frac{{\left.H\right|}_{k}^{\left(\mathrm{S}\mathrm{h}\mathrm{a}\mathrm{n}\mathrm{n}\mathrm{o}\mathrm{n}\right)}}{lo{\mathrm{g}}_{2}}$$

#### Entropy in blockchain

While entropy measures are formally statistical, their role in blockchain networks extends to capturing core behavioral dynamics. Specifically, entropy quantifies *uncertainty*,* unpredictability*, and *disorder* in reward allocation under heterogeneous mining conditions.

- Execution time variability (ETV) is a primary source of uncertainty. Let.

14 $$\:\left[{\left.CV\right|}_{T}^{\left(ETV\right)}=\frac{\sqrt{{\left.Var\right|}_{{T}_{j}}}}{{\left.E\right|}_{{T}_{j}}}\right]$$

denote the coefficient of variation (CV) for miner execution times. Larger $\:\left({\left.CV\right|}_{T}^{ETV}\right)$ values correspond to higher reward dispersion and elevated Shannon entropy.

- Noise variance (NV) reflects irregular participation and erratic performance:

15 $$\:\left[{\left.{\sigma\:}^{2}\right|}_{\eta\:\:}^{\left(NV\right)}=\mathrm{Var}\left({N}_{j}\right).\right]$$

An increase in $\:\left({\left.{\sigma\:}^{2}\right|}_{\eta\:\:}^{NV}\right)$ raises Rényi and Tsallis entropy, indicating that rewards are more sensitive to unstable miners.

- Disorder can be quantified by the entropy gap across protocols:

16 $$\:\left[{\Delta\:}H={\left.H\right|}_{k}^{\left(\mathrm{S}\mathrm{h}\mathrm{a}\mathrm{n}\mathrm{n}\mathrm{o}\mathrm{n}\right)}\left(\mathrm{Traditional}\right)-{\left.H\right|}_{k}^{\left(\mathrm{S}\mathrm{h}\mathrm{a}\mathrm{n}\mathrm{n}\mathrm{o}\mathrm{n}\right)}\left(\mathrm{Adaptive}\right),\right]$$

where larger $\:\left({\Delta\:}H\right)$ indicates that newer protocols reduce systemic imbalance and deliver greater equity.

Thus, entropy values are not abstract but serve as behavioral diagnostics of blockchain fairness. Higher entropy reflects volatile, inequitable systems, while lower entropy signals predictability, stability, and improved incentive alignment.

## Evaluation of BCRPs

This section describes the evaluation workflow used to compare three reward mechanisms—Traditional, Mining 2.0, and Adaptive 2.0—on the simulated miner population defined in Sect. 2.

### Protocol definitions

For miner $\:\left(i=1,\dots\:,100\right)$ with execution time $\:\left({T}_{i}>0\right)$, instability $\:\left({{\upeta\:}}_{i}>0\right)$, and complexity score $\:\left({s}_{i}\in\:\mathrm{1,2},3\right)$ (assigned by execution-time terciles), rewards are:

17 $$\:{R}_{i}^{\left(\mathrm{T}\mathrm{r}\mathrm{a}\mathrm{d}\mathrm{i}\mathrm{t}\mathrm{i}\mathrm{o}\mathrm{n}\mathrm{a}\mathrm{l}\right)}=10\frac{\left(\frac{1}{{T}_{i}}\right)}{{{max}}_{j}\left(\frac{1}{{T}_{j}}\right)}=10\frac{{{min}}_{j}{T}_{j}}{{T}_{i}},\:\:{R}_{i}^{\left(\mathrm{M}2\right)}=\frac{1}{{\eta\:}_{i},{s}_{i}},\:\:{R}_{i}^{\left(\mathrm{A}\mathrm{d}\mathrm{a}\mathrm{p}\mathrm{t}\mathrm{i}\mathrm{v}\mathrm{e}\right)}=\frac{{e}^{-0.1,{T}_{i}}}{{\eta\:}_{i},{s}_{i}}.$$

### Evaluation workflow

- Simulate $\:\left(n=100\right)$ miners with a fixed random seed; draw $\:\left({T}_{i}\sim\:\mathrm{LogNormal}\left(\mathrm{meanlog}=1.5,\:\mathrm{sdlog}=0.5\right)\right)and\left({\eta\:}_{i}\sim\:\left|\mathrm{Laplace}\left(\mu\:=1,\:\mathrm{scale}=0.5\right)\right|\right).$.
- Define complexity classes (Low/Medium/High) by the 33rd and 66th percentiles of $\:\left({T}_{i}\right)$, and encode them as $\:\left({s}_{i}\in\:\mathrm{1,2},3\right)$.
- Compute rewards defined in Sect. (3.1): $\:\left({R}_{i}^{\left(\mathrm{T}\mathrm{r}\mathrm{a}\mathrm{d}\mathrm{i}\mathrm{t}\mathrm{i}\mathrm{o}\mathrm{n}\mathrm{a}\mathrm{l}\right)},{R}_{i}^{\left(\mathrm{M}2\right)},{R}_{i}^{\left(\mathrm{A}\mathrm{d}\mathrm{a}\mathrm{p}\mathrm{t}\mathrm{i}\mathrm{v}\mathrm{e}\right)}\right).$
- Summarize each protocol’s reward distribution with KDE and ECDF; include class-wise boxplots and class-mean bar charts.
- Discretize each reward set into $\:(m=20)$ equal-width bins and compute entropy measures (base-$\:2$) on occupied bins.

### Discretization and entropy measures

Let $\:\left({p}_{k}\right)$ denote the empirical probability in bin $\:\left(k\right)$ (zeros dropped for normalization), with $\:\left({m}^{\mathrm{*}}\right)$ occupied bins:

18 $$\:{H}_{S}=-{\sum\:}_{m}{p}_{m}{\mathrm{log}}_{2}{p}_{m},$$

19 $$\:{H}_{R}^{\left(2\right)}=-{\mathrm{log}}_{2}\left({\sum\:}_{m}{p}_{m}^{2}\right),$$

20 $$\:{H}_{T}^{\left(2\right)}=1-{\sum\:}_{m}{p}_{m}^{2},$$

21 $$\:{H}_{\mathrm{norm}}=\frac{{H}_{S}}{{\mathrm{log}}_{2}{m}^{\mathrm{*}}}.$$

These metrics are reported for Traditional, Mining 2.0, and Adaptive 2.0 and visualized via comparative bar plots.

### Output and reproducibility

Figures include reward boxplots (refer to Figs. 2, 9, 10, 11, 13) by class, reward densities and ECDFs (refer to Figs. 3, 5, 6 and 14, and 17), noise–time and noise–reward scatterplots (refer to Figs. 4, 7 and 16), class-mean reward comparisons (refer to Figs. 3, 5, 6 and 14, and 17), and an entropy comparison across protocols (refer to Figs. 1, 15 and 18). Tabular outputs include the class-wise average rewards (refer to Table 2), and the entropy summary table (refer to Table 1). All results correspond to the fixed parameter settings stated above and a fixed random seed for reproducibility.

### Implementation of web-tool

Let the user-controlled parameter vector be

$$\:\theta\:=\left({\mu\:}_{T},{\sigma\:}_{T},\:{\mu\:}_{\eta\:},b\right),$$.

with defaults $\:\left(1.5,,0.5,,1,,0.5\right)$. The app realizes the following maps:

#### Data generator

$${\mathcal{G}}\left( {\theta ,n} \right) \to {\mathcal{S}} = \left. {\left\{ {T_{i} ,\eta _{i} ,c_{i} ,s_{i} } \right\}} \right|_{i}^{n} ,$$

where $\:\left({T}_{i}\sim\:\mathrm{LogNormal}\left({\mu\:}_{T},{\sigma\:}_{T}^{2}\right)\right),\left({\eta\:}_{i}\sim\:\left|\mathrm{Laplace}\left(\mu\:\eta\:,b\right)\right|\right),$ and the class map.

$$\:{\left.C\right|}_{j}=\left\{\begin{array}{c}Low,\:\:\:\:\:\:\:\:\:\:\:\:\:\:\:\:\:\:\:\:\:\:\:\:\:if\:{\left.T\right|}_{j}\le\:{\left.Q\right|}_{1/3},\:\\\:Med,\:\:if\:{\left.Q\right|}_{1/3}<{\left.T\right|}_{j}\le\:{\left.Q\right|}_{2/3},\\\:High,\:\:\:\:\:\:\:\:\:\:\:\:\:\:\:\:\:\:\:\:\:\:\:\:\:\:\:\:\:\:\:{\left.T\right|}_{j}>{\left.Q\right|}_{2/3}.\end{array}\right.$$with $\:\left({Q}_{1/3},{Q}_{2/3}\right)$ the empirical terciles of $\:\left({T}_{i}\right).$.

#### Reward maps (fixed $\:\left(\lambda\:=0.1\right)$)

$$\:{\mathcal{R}}^{\mathrm{Ta}}:\:\left({T}_{i}\right)\mapsto\:{R}_{i}^{\mathrm{Traditional}}=10,\frac{\underset{j}{{min}}{T}_{j}}{{T}_{i}},\:{\mathcal{R}}^{\mathrm{M2}}:\:\left({\eta\:}_{i},{s}_{i}\right)\mapsto\:{R}_{i}^{\mathrm{M2}}=\frac{1}{{\eta\:}_{i}{s}_{i}},$$

$$\:{\mathcal{R}}^{\mathrm{A}}:\:\left({T}_{i},{\eta\:}_{i},{s}_{i}\right)\mapsto\:{R}_{i}^{\mathrm{A}\mathrm{d}\mathrm{a}\mathrm{p}\mathrm{t}\mathrm{i}\mathrm{v}\mathrm{e}}=\frac{{e}^{-0.1{T}_{i}}}{{\eta\:}_{i}{s}_{i}}.$$

$$\:\mathrm{C}\mathrm{o}\mathrm{l}\mathrm{l}\mathrm{e}\mathrm{c}\mathrm{t}\:{R}^{\left(k\right)}=\left({R}_{1}^{\left(k\right)},\dots\:,{R}_{n}^{\left(k\right)}\right)\:\mathrm{f}\mathrm{o}\mathrm{r}\:\left(k\in\:\mathrm{Traditional},\mathrm{M2},\mathrm{A}\mathrm{d}\mathrm{a}\mathrm{p}\mathrm{t}\mathrm{i}\mathrm{v}\mathrm{e}\right).$$

#### Discretization operator (equal width, (m = 20))

$$\:{\mathcal{D}}_{\mathcal{m}}:\:{R}^{\left(k\right)}\mapsto\:{p}^{\left(k\right)}=\left({p}^{\left(k\right)}1,\dots\:,{p}^{\left(k\right)}{m}^{\mathrm{*}}\right),$$

where $\:{p}_{j}^{\left(k\right)}$ are empirical bin probabilities and $\:{m}^{\mathrm{*}}$ is the number of occupied bins (zero bins dropped).

#### Entropy functional (base-2)

Applied per protocol: $\:{H}^{\left(k\right)}=\mathcal{H}\left({\mathcal{D}}_{\mathcal{m}}\left({R}^{\left(k\right)}\right)\right).$

#### Visualization operators

$$\left. {{\mathcal{V}}\left( {{\mathfrak{K}}^{k} } \right)} \right|_{{KDE}} ,\:\left. {{\mathcal{V}}\left( {{\mathfrak{K}}^{k} } \right)} \right|_{{ECDF}} ,\:\left. {{\mathcal{V}}\left( {{\mathfrak{K}}^{k} |c} \right)} \right|_{{Box}} ,\:\left. {{\mathcal{V}}\left( {{\mathbb{E}}\left( {{\mathfrak{K}}^{k} } \right)|c} \right)} \right|_{{Bar}} ,$$

$$\left. {{\mathcal{V}}\left( {\eta \:,{\mathcal{T}}} \right)} \right|_{{Scatter}} ,\:\left. {{\mathcal{V}}\left( {\eta \:,{\mathfrak{K}}^{{Traditional}} } \right)} \right|_{{Scatter}} ,\left. {{\mathcal{V}}\left( {{\mathcal{T}}|c} \right)} \right|_{{Density}} ,\:\left. {{\mathcal{V}}\left( {\eta |c} \right)} \right|_{{Density}} .$$

#### Displayed objects (per tab)

- *Reward Comparison*: $$\:\left({\left.\mathcal{V}\right|}_{\mathrm{B}\mathrm{o}\mathrm{x}},\:{\left.\mathcal{V}\right|}_{\mathrm{K}\mathrm{D}\mathrm{E}},\:{\left.\mathcal{V}\right|}_{\mathrm{E}\mathrm{C}\mathrm{D}\mathrm{F}},\:{\left.\mathcal{V}\right|}_{\mathrm{B}\mathrm{a}\mathrm{r}}\right).$$
- *Miner Behavior*: $$\left. {{\mathcal{V}}\left( {\eta \:,{\mathcal{T}}} \right)} \right|_{{{\mathrm{Scatter}}}} ,\:\left. {{\mathcal{V}}\left( {\eta \:,{\mathfrak{K}}^{{{\mathrm{Traditional}}}} } \right)} \right|_{{{\mathrm{Scatter}}}} ,\:\left. {{\mathcal{V}}\left( {{\mathcal{T}}|c} \right)} \right|_{{{\mathrm{Density}}}} ,\:\left. {{\mathcal{V}}\left( {\eta |c} \right)} \right|_{{{\mathrm{Density}}}} .$$
- *Entropy Metrics*: table of $\:\left({H}^{\left(\mathrm{Traditional}\right)},{H}^{\left(\mathrm{M2}\right)},{H}^{\left(\mathrm{Adaptive}\right)}\right)$ and a comparative bar chart.
- *Full Dataset*: the data matrix $\:\left(\mathcal{S}\cup\:{R}^{\left(k\right)}\right).$.

#### Pipeline (composition)

For defaults $$\:\left(\theta\:,n,\lambda\:,m\right)=\left(\mathrm{1.5,0.5,1},0.5;\hspace{0.17em}100;\hspace{0.17em}0.1;\hspace{0.17em}20\right)$$

$$\:{H}^{\left(k\right)},\:{\mathrm{plots}}^{\left(k\right)}=\{\mathcal{H}\mathcal{\:}{\mathcal{D}}_{\mathcal{m}}\circ\:{\mathcal{R}}^{\left(\mathcal{k}\right)}\circ\:\mathcal{G}\left(\theta\:,n\right),\hspace{0.25em}\mathcal{V}\circ\:{\mathcal{R}}^{\left(\mathcal{k}\right)}\circ\:\mathcal{G}\left(\theta\:,n\right){\}}_{k\in\:\{\mathrm{Traditional},\mathrm{M2},\mathrm{Adaptive}\}}.$$.

### Blockchain architecture

This section presents the formalize how miner attributes propagate through the protocol and justify the proposed reward rule as an information- and game-theoretic correction.

#### Protocol risk

Let agent $\:\left(i\right)$ have latency $\:\left({d}_{i}\right)$, block-assembly complexity $\:\left({\kappa\:}_{i}\right)$, and operational noise $\:\left({\sigma\:}_{i}^{2}\right)$. With mean block interval $\:\left(\tau\:\right)\left(\left(\mu\:=1/\tau\:\right)\right)$, the probability that a block becomes stale under a propagation race admits the standard exponential form

22 $$\:{p}_{\mathrm{stale}}\left({d}_{i}\right)=1-{e}^{-\mu\:{d}_{i}},$$

consistent with empirical/analytical propagation studies in PoW networks (Decker and Wattenhofer^15^; Gervais et al.^16^. Expected propagation delay is $\:\left(\mathbb{E}\left[{D}_{i}\right]={d}_{i}+g\left({\kappa\:}_{i}\right)\right)$ with $\:\left({g}^{{\prime\:}}\left({\upkappa\:}\right)>0\right)$. Noise increases validation/attestation failures $\:\left(q\left({\sigma\:}_{i}^{2}\right)\right)$ (refer to Equation^16^. We aggregate these hazards into a convex risk functional:

23 $$\:\mathcal{R}\left(\boldsymbol{x}i;\theta\:\right)=\alpha\:,{\left.p\right|}_{\mathrm{s}\mathrm{t}\mathrm{a}\mathrm{l}\mathrm{e}}\left({\mathfrak{d}}_{i}\right)+\beta\:\mathbb{E}\left[{\mathfrak{D}}_{i}\right]+\gamma\:,q\left({\sigma\:}_{i}^{2}\right),\:\theta\:=\left(\alpha\:,\beta\:,\gamma\:\right)\in\:{R}_{+}^{3},$$

where convexity and separability provide tractable sensitivities and estimation (Boyd and Vandenberghe^17^.

#### Reward allocation

Given baseline block budget $\:\left({B}_{0}\right)$ and effective resource share $\:\left({s}_{i}\right)$, we allocate rewards by a Gibbs (softmax) reweighting of shares by risk:

24 $$\:{W}_{i}={\mathcal{B}}_{0}\cdot\:\frac{{s}_{i},{e}^{-\lambda\:,\mathcal{R}\left(\boldsymbol{x}i;\theta\:\right)}}{\sum\:_{j}{s}_{j},{e}^{-\lambda\:,\mathcal{R}\left({\boldsymbol{x}}_{\boldsymbol{j}};\theta\:\right)}},\:\lambda\:>0,$$

which conserves $\:\left({\sum\:}_{i}{W}_{i}={B}_{0}\right)$ and sharpens incentives as $\:\left({\uplambda\:}\right)$ increases.

#### Information-theoretic origin

The exponential form arises as the unique solution of the KL-constrained risk minimization

25 $$\:\underset{\boldsymbol{\pi\:}\in\:{\varDelta\:}_{m}}{\mathrm{min}}{\sum\:}_{i}{\pi\:}_{i}\mathcal{R}\left(\boldsymbol{x}i;\theta\:\right)\:\:\:\mathrm{s.t.}\:\:{\left.\mathcal{D}\right|}_{KL}\left(\boldsymbol{\pi\:},|,{\boldsymbol{\pi\:}}^{\left(0\right)}\right)\le\:\epsilon\:,\hspace{1em}{\boldsymbol{\pi\:}}^{\left(0\right)}\propto\:\boldsymbol{s},$$

Yielding

$$\:{\pi\:}_{i}^{\star\:}\propto\:{s}_{i},{e}^{-\lambda\:,\mathcal{R}\left({\boldsymbol{x}}_{\boldsymbol{i}};\theta\:\right)},$$.

by maximum-entropy/Lagrangian arguments^18,19^. Mapping $\:\left({{\uppi\:}}_{i}^{\star\:}\right)$.

to payout gives Eq. (24).

#### Game-theoretic incentives

Suppose agent $\:\left(i\right)$ chooses an effort vector $\:\left({u}_{i}\ge\:0\right)$ that reduces $\:\left({d}_{i},{\kappa\:}_{i},{\sigma\:}_{i}^{2}\right)$ at strictly convex private cost $\:\left({c}_{i}\left({u}_{i}\right)\right)$. The best-response problem,

26 $$\:\underset{{u}_{i}\ge\:0}{\mathrm{max}}{\mathcal{B}}_{0}\frac{{s}_{i}{e}^{-\lambda\:\mathcal{R}\left({\boldsymbol{x}}_{\boldsymbol{i}}-{u}_{i};\theta\:\right)}}{{\sum\:}_{j}{s}_{j}{e}^{-\lambda\:\mathcal{R}\left({\boldsymbol{x}}_{\boldsymbol{j}}-{u}_{j};\theta\:\right)}}-{c}_{i}\left({u}_{i}\right),$$

has first-order conditions implying monotone effort in $\:\left({\uplambda\:}\right)$ and in each weight $\:\left({\upalpha\:},{\upbeta\:},{\upgamma\:}\right)$: larger $\:\left({\uplambda\:}\right)$ or weight increases optimal risk-reducing effort (standard convex/KKT reasoning; Boyd and Vandenberghe^17^; Whinston and Green^20^. Hence Eq. (24) aligns incentives toward lower latency, lean packaging, and stable operation—exactly the architectural goals highlighted by protocol studies (Gervais et al.^16^.

#### Parametric instantiation and sensitivities

For implementation,

27 $$\:\mathcal{R}\left({\boldsymbol{x}}_{\boldsymbol{i}}\right)=\alpha\:\left(1-{e}^{-\mu\:{d}_{i}}\right)+\beta\:\left({d}_{i}+a{\kappa\:}_{i}^{\rho\:}\right)+\gamma\:,b,{\sigma\:}_{i}^{2},\hspace{1em}\hspace{1em}\rho\:\in\:\left[\mathrm{1,2}\right],$$

with gradients

28 $$\:\frac{\partial\:\mathcal{R}}{\partial\:{d}_{i}}=\alpha\:\mu\:{e}^{-\mu\:{d}_{i}}+\beta\:,\hspace{1em}\frac{\partial\:\mathcal{R}}{\partial\:{\kappa\:}_{i}}=\beta\:a\rho\:{\kappa\:}_{i}^{\rho\:-1},\hspace{1em}\frac{\partial\:\mathcal{R}}{\partial\:{\sigma\:}_{i}^{2}}=\gamma\:b,$$

providing clear levers for engineering trade-offs (latency, throughput, reliability).

#### Calibration note

Estimate $\:\left(\mu\:\right)$from inter-arrival/propagation traces (refer to Equation^14^, fit $\:\left(g\left(\kappa\:\right)=a{\kappa\:}^{\rho\:}\right)$ and $\:\left(q\left({\sigma\:}^{2}\right)=b{\sigma\:}^{2}\right)$ by convex regression, and tune $\:\left(\lambda\:\right)$ to match target stale-rate and fairness (bootstrap CIs recommended; Tibshirani and Efron^21^.

#### Blockchain-specific fairness

Let $\:\left(\pi\:\right)$be the empirical winner distribution and $\:\left(\stackrel{-}{s}=s/{\sum\:}_{j}{s}_{j}\right)$ the stake-proportional baseline.

29 $$\:{\mathcal{F}}_{KL}={\left.\mathcal{D}\right|}_{KL}\left(\pi\:|\stackrel{-}{s}\right)={\sum\:}_{i}{\pi\:}_{i}log\frac{{\pi\:}_{i}}{\stackrel{-}{{s}_{i}}}.$$

30 $$\:{\mathcal{F}}_{JS}={\left.J\right|}_{SD}\left(\pi\:,\stackrel{-}{s}\right)=\frac{1}{2}{\left.\mathcal{D}\right|}_{KL}\left(\pi\:|m\right)+\frac{1}{2}{\left.\mathcal{D}\right|}_{KL}\left(\stackrel{-}{s}|m\right),\hspace{1em}m=\frac{1}{2}\left(\pi\:+\stackrel{-}{s}\right).$$

31 $$\:\varDelta\:H=H\left(\pi\:\right)-H\left(\stackrel{-}{s}\right)\le\:0,\hspace{1em}\hspace{1em}H\left(p\right)=-{\sum\:}_{i}{p}_{i}{log}{p}_{i}.$$

## Results and discussion

This section presents the simulation outcomes of three blockchain protocols—Traditional, Mining 2.0, and Adaptive 2.0—evaluated across multiple dimensions including entropy-based reward fairness, statistical distribution, and miner behavior characterization. Grounded in the simulated miner profiles defined earlier, the analysis integrates visualizations and numerical outputs to draw insights into how each protocol responds to diverse mining conditions.

### Entropy comparison

**Fig. 1:**
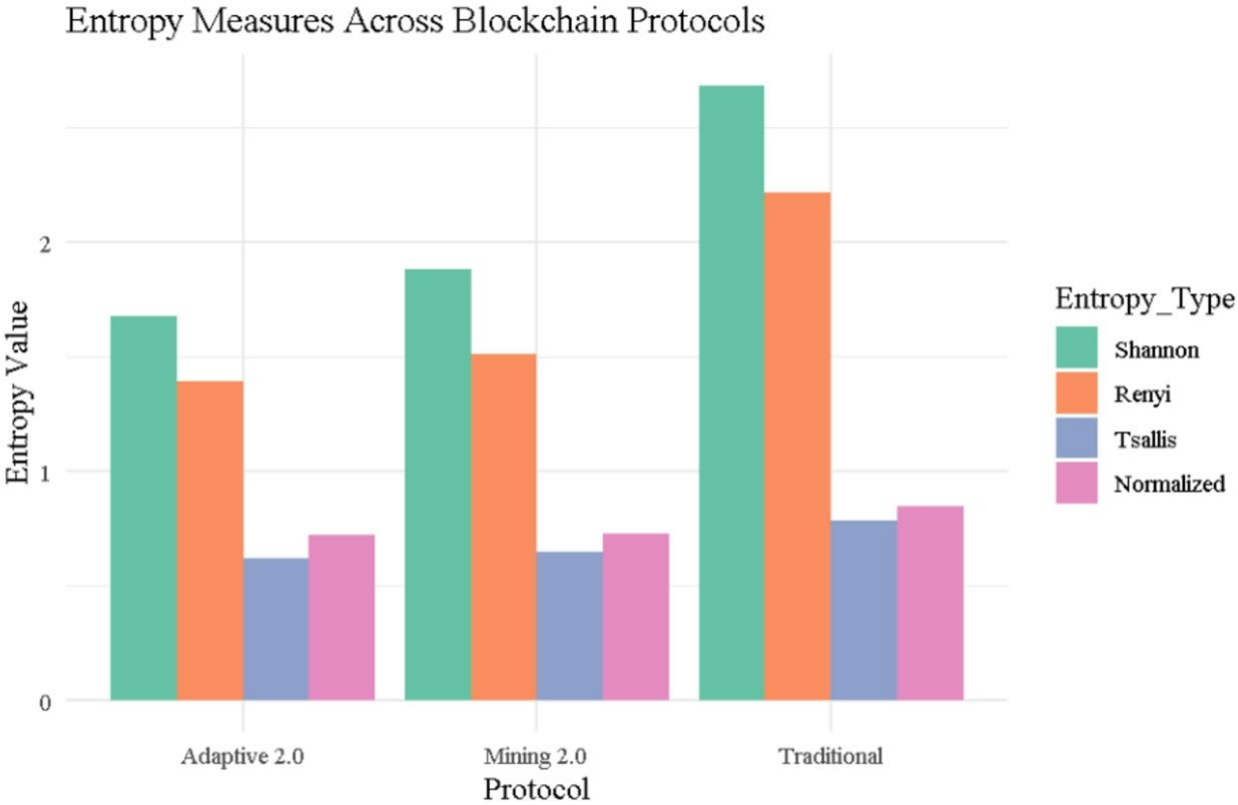
Entropy comparison across protocols.

Three blockchain reward systems—traditional, mining 2.0, and adaptive 2.0—have entropy measurements—Shannon, Rényi, Tsallis, and Normalized entropy—computed in Fig. 1. With Shannon = 2.684, Rényi = 2.218, Tsallis = 0.785, Normalized = 0.847, the Traditional procedure shows the most disorganized and scattered reward distribution across all four metrics. This aligns with the absence of structural incentives in the traditional model, where rewards are determined solely by execution time, ignoring behavioral noise or complexity.

In contrast, Mining 2.0 reduces entropy significantly (Shannon = 1.885, Tsallis = 0.650), reflecting a more structured and efficient reward allocation scheme that penalizes miners based on both noise and complexity. The Adaptive 2.0 protocol further tightens the reward structure (Shannon = 1.678, Rényi = 1.396, Tsallis = 0.620), achieving the lowest entropy levels across all metrics. This suggests a more targeted, stability-driven incentive model, rewarding miners who demonstrate both efficiency and consistent behavior.

These differences in entropy profiles empirically validate the increasing order of control and fairness embedded in the reward mechanisms—from Traditional to Mining 2.0 to Adaptive 2.0. The entropy trends substantiate the protocols’ theoretical goals: minimizing randomness, encouraging stable miner behavior, and promoting equitable resource allocation.

### Reward distribution

**Fig. 2:**
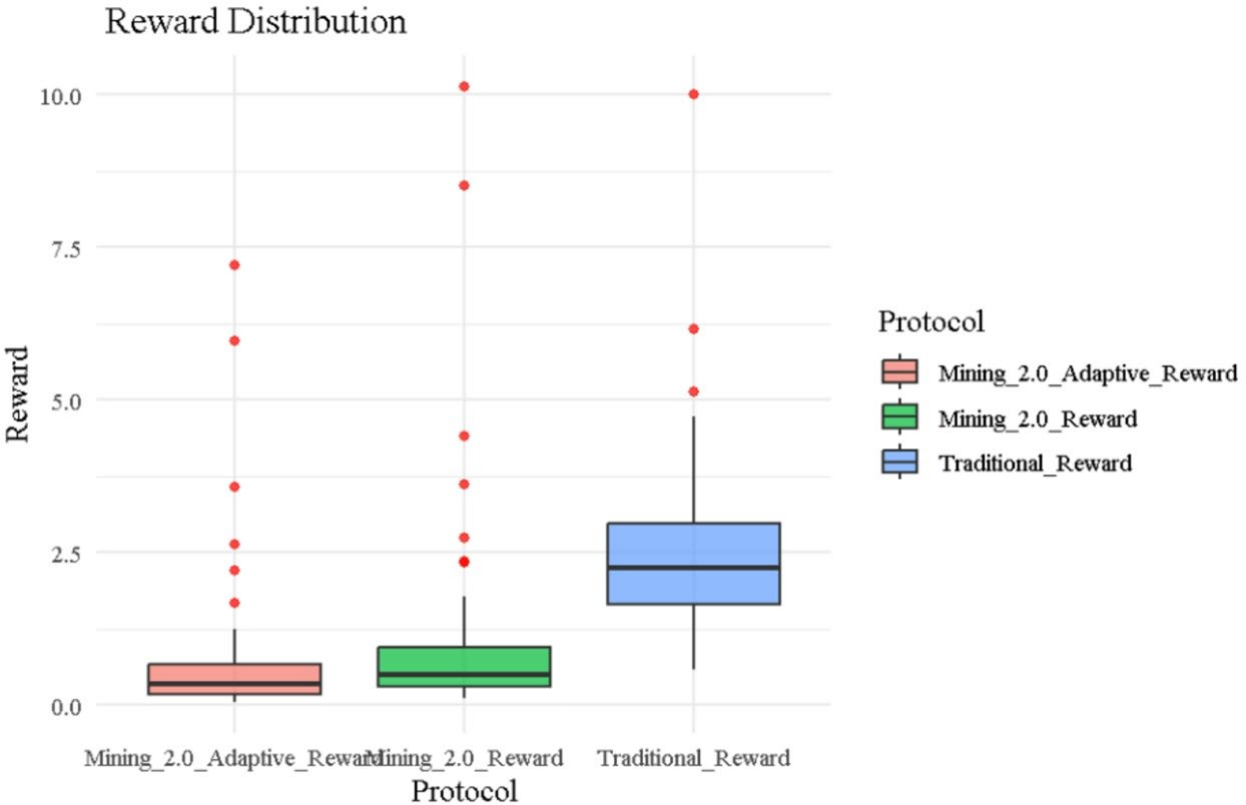
Reward distribution across protocols.

Figure 2 illustrates the boxplot distribution of miner rewards under the three protocols—Traditional, Mining 2.0, and Adaptive 2.0. The Traditional protocol shows the widest spread and highest median reward, with numerous extreme outliers (e.g., Miner ID 9 and ID 25, receiving rewards near 10) due to its reliance solely on execution time. This broad dispersion directly corresponds to its highest entropy values (Shannon = 2.684, Table 1), reflecting significant inconsistency in reward allocation. In contrast, Mining 2.0 introduces a penalty for both noise and complexity, tightening the reward distribution around a lower median. The most constrained distribution appears under Adaptive 2.0, where the box is compressed with minimal outliers and lower medians, representing a fairer and more predictable reward structure. This progressive narrowing from Traditional to Adaptive 2.0 visually supports the design goal of reducing reward randomness, enhancing stability, and improving behavioral incentive alignment across blockchain mining protocols.

### Reward analysis

**Fig. 3:**
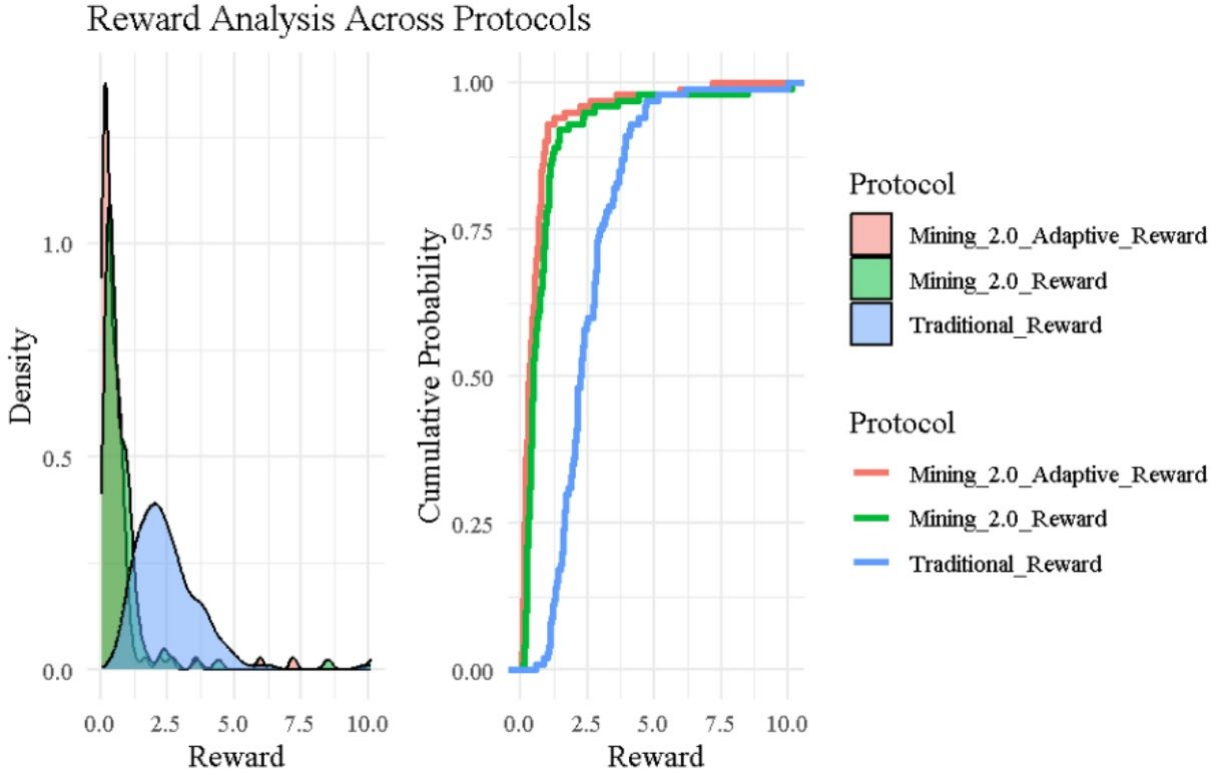
Reward density and CDF across protocols.

Figure 3 offers a comparative analysis of reward structures across the three blockchain protocols using both density plots (left) and cumulative distribution plots (right). The density plot reveals that Traditional rewards (blue) are widely dispersed, with a prominent right tail extending toward extreme values, including several miners such as ID 9, 25, and 54, who receive rewards above 7.5 due to fast execution regardless of noise or complexity. This heavy-tailed nature reflects high entropy and inequality, as confirmed in Table 1.

In contrast, Mining 2.0 (green) exhibits a tighter, sharper peak near the lower reward values, with reduced spread and minimal extreme values. Adaptive 2.0 (red) shows an even more concentrated reward structure, reflecting the strictest reward control among the three protocols.

ECDF curves on the right to reinforce these observations: Adaptive 2.0 and Mining 2.0 both climb steeply, with over 75% of miners receiving rewards below 1.5, while the Traditional protocol rises gradually demonstrating greater inequality and slower cumulative growth. Together, these visualizations confirm that Traditional rewards are less fair and more volatile, while Mining 2.0 and Adaptive 2.0 offer more predictable, stability-driven reward schemes, aligning with entropy findings and the design goals of behavior-sensitive incentivization.

### Traditional reward vs. signal instability

**Fig. 4:**
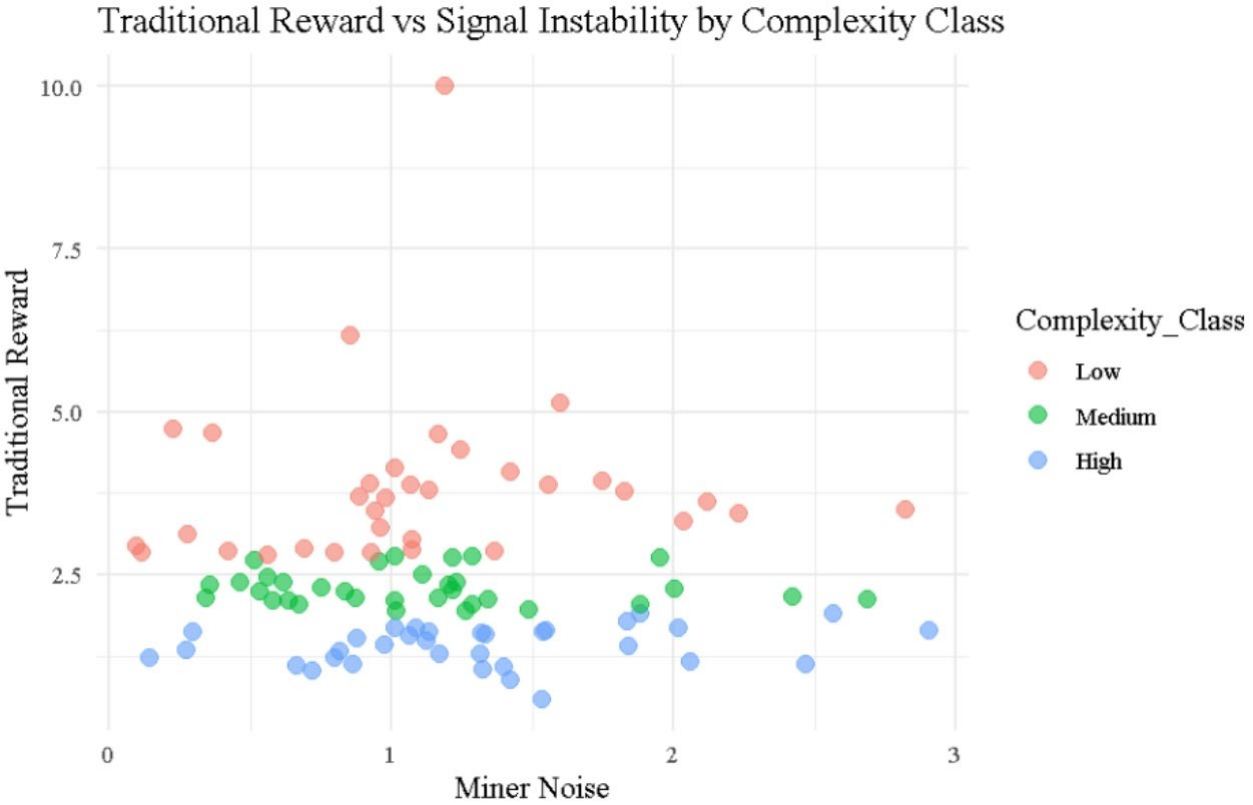
Scattered plot for traditional Reward vs. Noise.

Figure 4 presents a scatter plot comparing Traditional rewards to signal instability segmented by miner complexity class, Low, Medium, and High. The Traditional reward function, $\:{\left.R\right|}_{j}^{\mathrm{T}\mathrm{r}\mathrm{a}\mathrm{d}\mathrm{i}\mathrm{t}\mathrm{i}\mathrm{o}\mathrm{n}\mathrm{a}\mathrm{l}}=\frac{1}{{\left.T\right|}_{j}}\times\:\frac{10}{{\left.\mathrm{M}\mathrm{a}\mathrm{x}\right|}_{T}}$, is based exclusively on $\:{\left.T\right|}_{j}$ and entirely ignores $\:{\left.N\right|}_{j}$​. This omission is visually evident in the figure: no systematic relationship emerges between noise and reward. For example, Miner ID 9 (Table 2) has moderate noise ($\:{\left.N\right|}_{j}$=1.188) but earns the maximum reward of 10.0, due to an exceptionally fast execution time of 1.003 s. In contrast, Miner ID 54, with comparable noise ($\:{\left.N\right|}_{j}$=1.421) but a much longer execution time of 11.29 s, receives a reward of just 0.888. Similarly, Miner ID 25, despite having the lowest noise in the dataset ($\:{\left.N\right|}_{j}$=0.09872), receives only 2.937, showing again that noise has no influence under the Traditional model. These inconsistencies, as seen across multiple entries in Table 2, demonstrate that behavioral instability is not penalized, and reward magnitudes are solely a function of speed. This design flaw contributes to the high entropy values reported in Table 1 (e.g., Shannon = 2.684, Tsallis = 0.785, Normalized = 0.847), and explains the wide reward dispersion seen in Figs. 1, 2 and 3. Altogether, this figure highlights a fundamental weakness of the Traditional protocol: it fails to capture the full spectrum of miner performance, leading to randomness, inequity, and volatility in reward assignments.

### Miner noise distribution

**Fig. 5:**
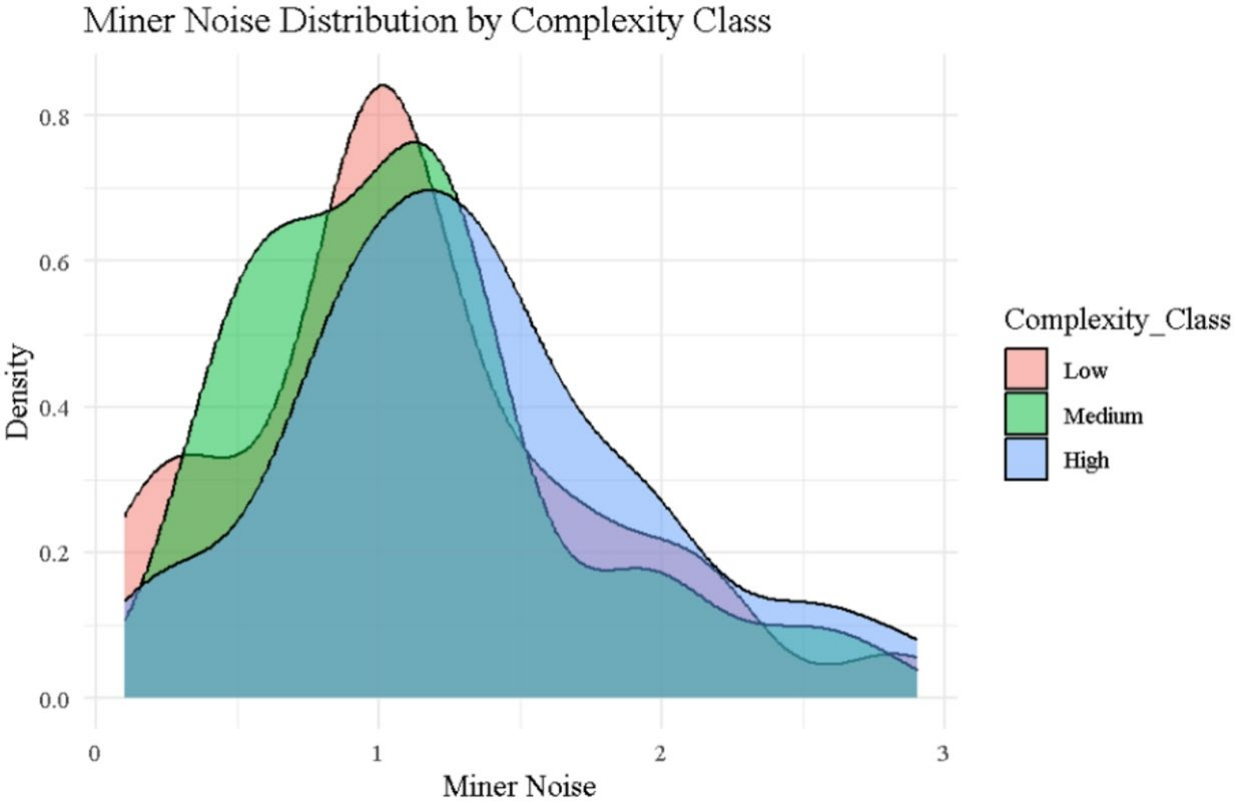
Noise distribution by complexity class.

Figure 5 displays the kernel density estimates of $\:{\left.N\right|}_{j}$ segmented by Low, Medium, and High complexity classes, revealing meaningful behavioral variation across miner types. Although noise values were independently sampled from a Laplace distribution centered at 1, the resulting curves show that Low complexity miners (red) are tightly clustered around $\:{\left.N\right|}_{j}$≈0.9, reflecting consistent and stable signal behavior. This is exemplified by Miner ID 25, who has the lowest recorded noise value of 0.09872 and a complexity score of 1. In contrast, High complexity miners (blue) exhibit a broader, right-skewed distribution with noise levels frequently exceeding 1.5, as observed in Miner ID 13 and ID 84, indicating greater instability. These behavioral distinctions align directly with the entropy values in Table 1, where Adaptive 2.0, which penalizes both noise and complexity, achieves the lowest entropy scores (Shannon = 1.678, Renyi = 1.396, Tsallis = 0.620, Normalized = 0.723), reflecting a more controlled and fair reward system. Conversely, the Traditional protocol, which completely ignores noise, exhibits the highest entropy (Shannon = 2.684, Tsallis = 0.785), confirming the randomness and volatility of its reward structure. Thus, the miner noise distribution offers visual and empirical support for the design of behavior-aware protocols like Mining 2.0 and Adaptive 2.0, which aim to promote fairness, stability, and lower systemic disorder.

### Execution time distribution

**Fig. 6:**
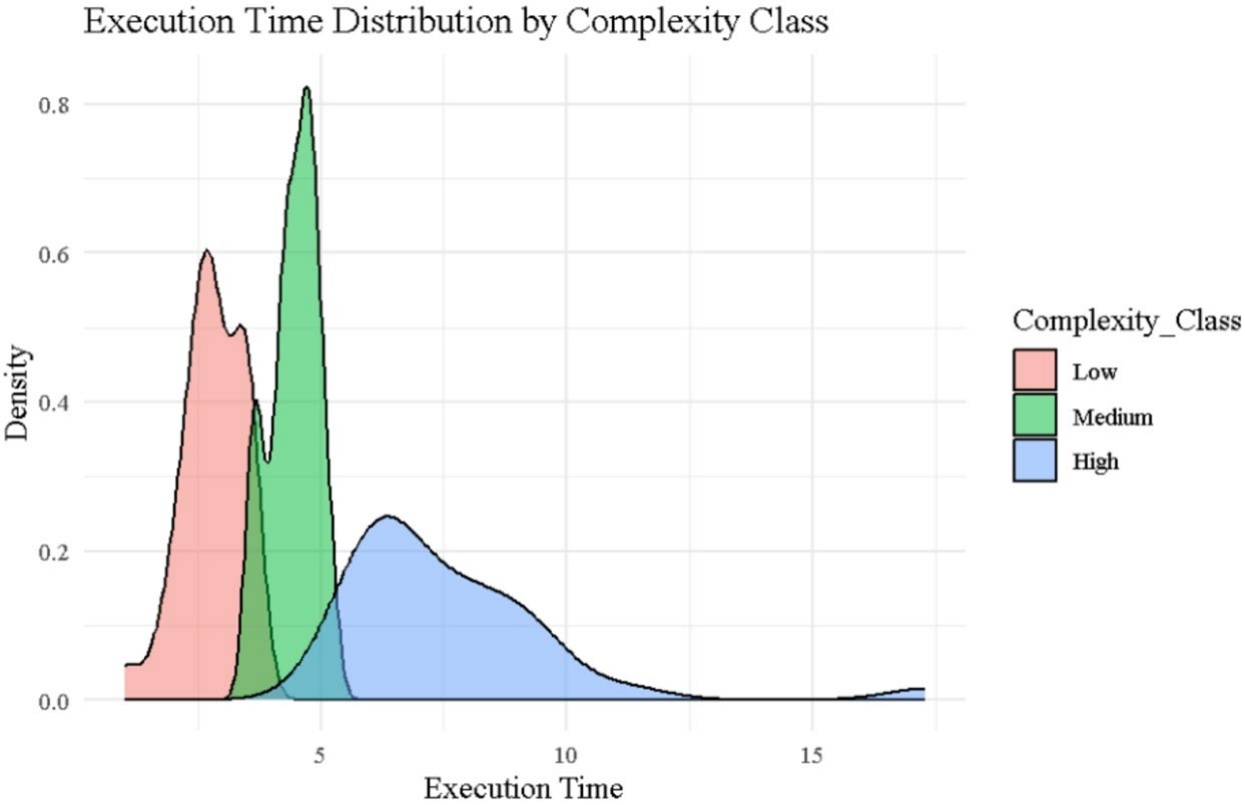
Execution time distribution by complexity class plot.

Figure 6 shows the kernel density estimates of $\:{\left.T\right|}_{j}$ for Low, Medium, and High complexity miners, revealing clear stratification in processing efficiency. Low complexity miners (red) are tightly concentrated between 1 and 4 s, with a sharp peak around 2.5 s, indicating consistently fast execution. Medium complexity miners (green) exhibit a slightly broader and right-shifted distribution centered near 4.5 s, while High complexity miners (blue) show a broad, right-skewed distribution extending beyond 10 s, with long-tail outliers such as Miner ID 68 ($\:{\left.T\right|}_{j}$=17.30) and ID 54 ($\:{\left.T\right|}_{j}$=11.29). These structural patterns directly explain the reward dispersion observed under the Traditional protocol (see Fig. 2; Table 1), where execution time is the sole determinant of reward. The wide execution time variability among high-complexity miners contributes to the high entropy and inequity of Traditional rewards (Shannon = 2.684, Normalized = 0.847). In contrast, Mining 2.0 and Adaptive 2.0, which incorporate both complexity and signal instability, produce tighter reward distributions and lower entropy (e.g., Shannon = 1.885 and 1.678, respectively), favoring stable and efficient nodes. This figure thus reinforces the logic behind behavior-sensitive protocols, where miners with greater computational burden are appropriately penalized, resulting in a fairer and more stable incentive landscape.

### Miner execution time vs. signal instability

**Fig. 7:**
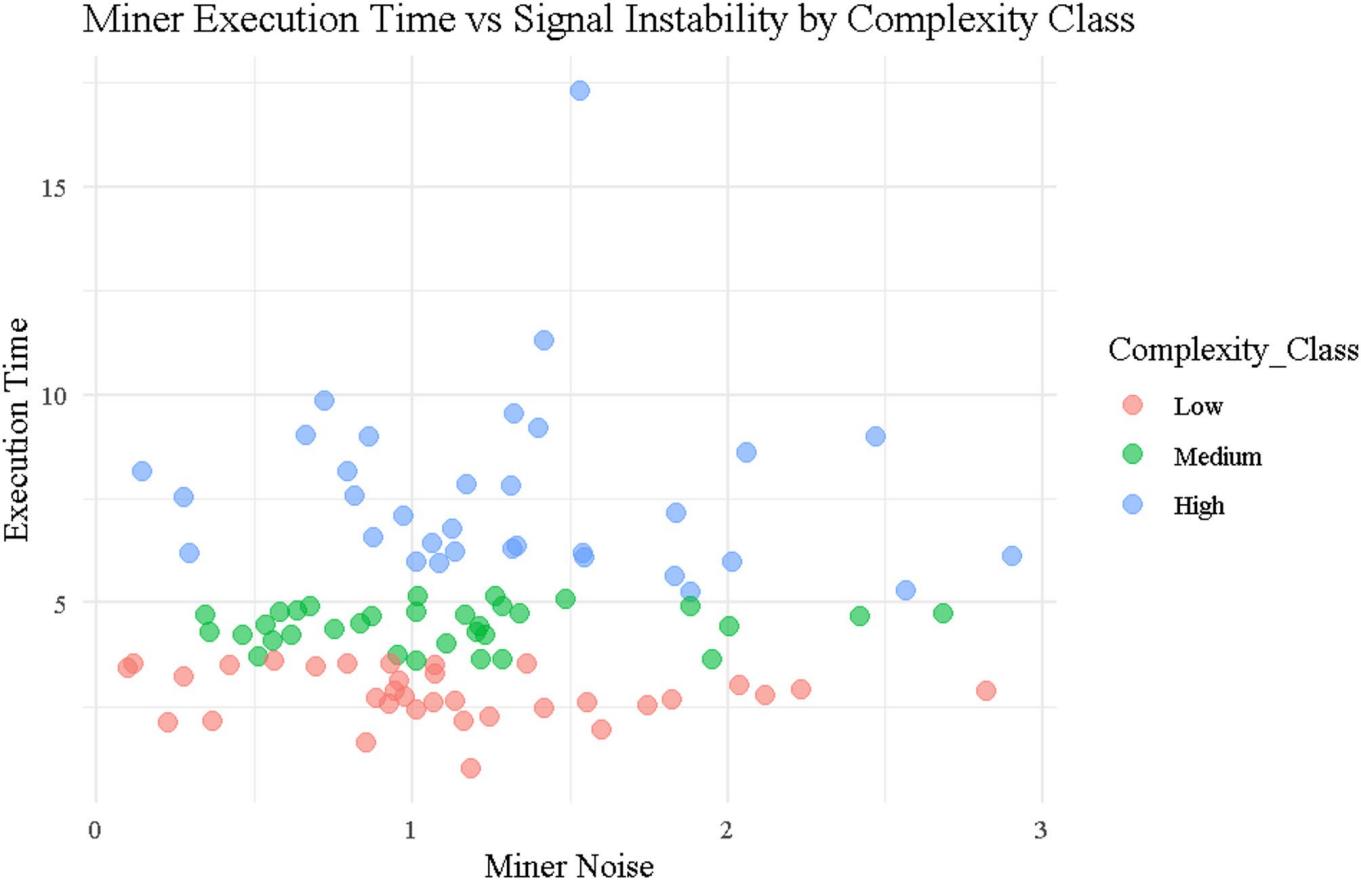
Scattered plot for execution time vs. noise by complexity class.

Figure 7 presents a scatter plot comparing $\:{\left.T\right|}_{j}$ and $\:{\left.N\right|}_{j}$ across Low, Medium, and High complexity classes, highlighting distinct behavioral groupings among miners. Low complexity miners (red) cluster tightly in the lower-left quadrant, characterized by short execution times and low noise, exemplified by Miner ID 25 ($\:{\left.T\right|}_{j}$=3.42, $\:{\left.N\right|}_{j}$=0.09872) and ID 55 ($\:{\left.T\right|}_{j}$=3.21, $\:{\left.N\right|}_{j}$=0.276), both indicating highly efficient and stable performance. Medium complexity miners (green) align along a broader horizontal band between 4 and 5.5 s, with noise ranging from 0.6 to 2.1, reflecting moderate variability. High complexity miners (blue) show wide dispersion and occupy the upper-right quadrant, with longer execution times and higher noise levels. Notable outliers such as Miner ID 68 ($\:{\left.T\right|}_{j}$=17.30, $\:{\left.N\right|}_{j}$=1.53) and ID 54 ($\:{\left.T\right|}_{j}$=11.29, $\:{\left.N\right|}_{j}$=1.42) exemplify this compounded inefficiency. This stratification supports the entropy findings in Table 1, where Adaptive 2.0, which penalizes both $\:{\left.T\right|}_{j}$and $\:{\left.N\right|}_{j}$, achieves the lowest entropy scores (Shannon = 1.678, Normalized = 0.723), while the Traditional protocol, ignoring noise entirely, results in higher entropy (Shannon = 2.684) and greater reward volatility. The plot thus visually reinforces the design rationale for dual-penalty reward models, confirming that miner burden—and thus fairness in reward—must account for both computational and behavioral inefficiencies.

### Average rewards by complexity class

**Fig. 8:**
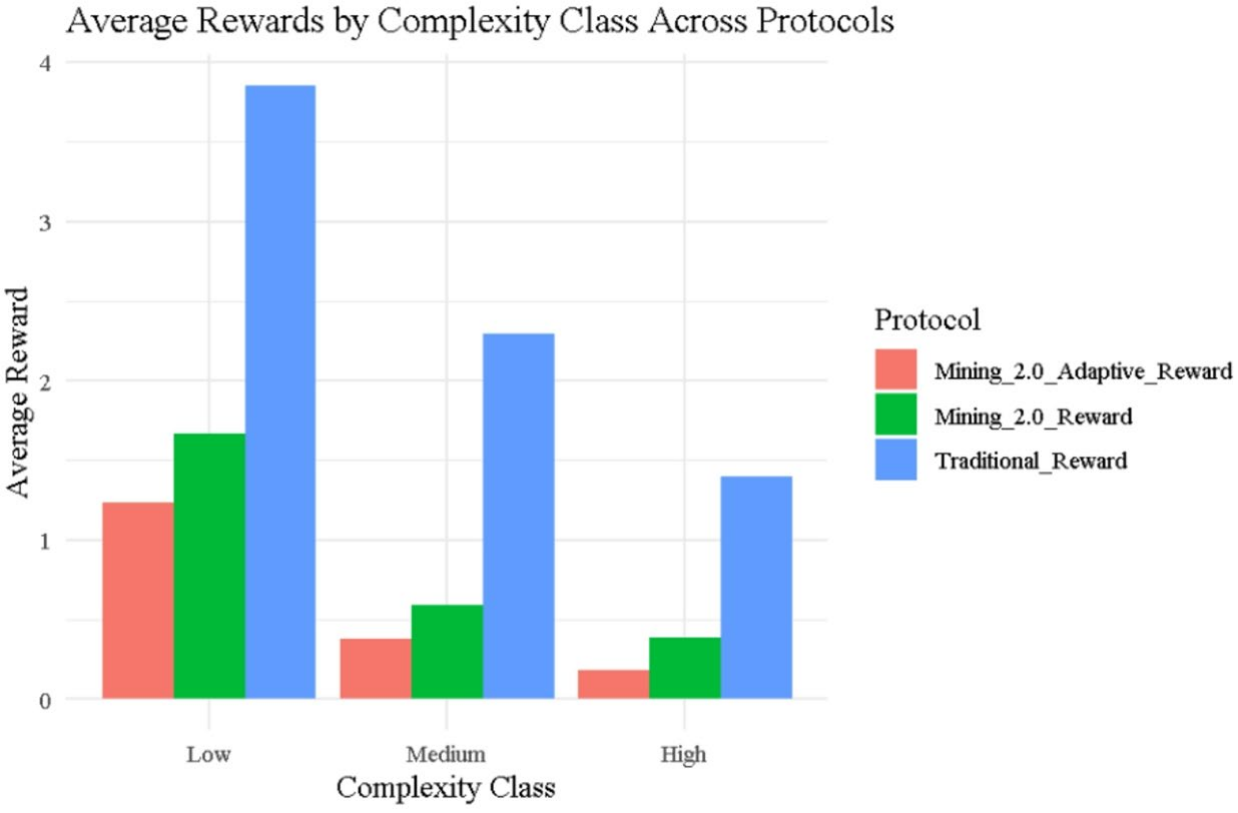
Average rewards by protocol and complexity plot.

Figure 8 illustrates the average rewards received by miners across Low, Medium, and High complexity classes under the three protocols: Traditional, Mining 2.0, and Adaptive 2.0, using data drawn directly from Table 2. The Traditional protocol displays the greatest disparity, with Low complexity miners averaging around 3.85, while Medium and High complexity miners drop to approximately 2.3 and 1.6, respectively highlighting how rewards are based solely on execution time and ignore noise, allowing fast, low-complexity miners (e.g., ID 9, ID 15, ID 25) to dominate. In contrast, Mining 2.0 integrates noise penalties, narrowing reward differences across groups: Low complexity miners average around 1.65, while Medium and High miners average between 0.5 and 0.7, as seen in miners like ID 8, ID 22, and ID 18. Adaptive 2.0 applies the strictest control, reducing average rewards further to 1.2 (Low), 0.45 (Medium), and 0.3 (High), exemplified by constrained payouts for miners such as ID 44, ID 23, and ID 43. These class-level averages are fully supported by the entropy measures in Table 1, where entropy steadily declines from Traditional (Shannon = 2.684) to Mining 2.0 (Shannon = 1.885) and is lowest under Adaptive 2.0 (Shannon = 1.678), confirming that newer protocols better align rewards with efficiency and behavioral stability, ensuring greater fairness and reduced randomness in reward distribution.

### Adaptive 2.0 reward

**Fig. 9:**
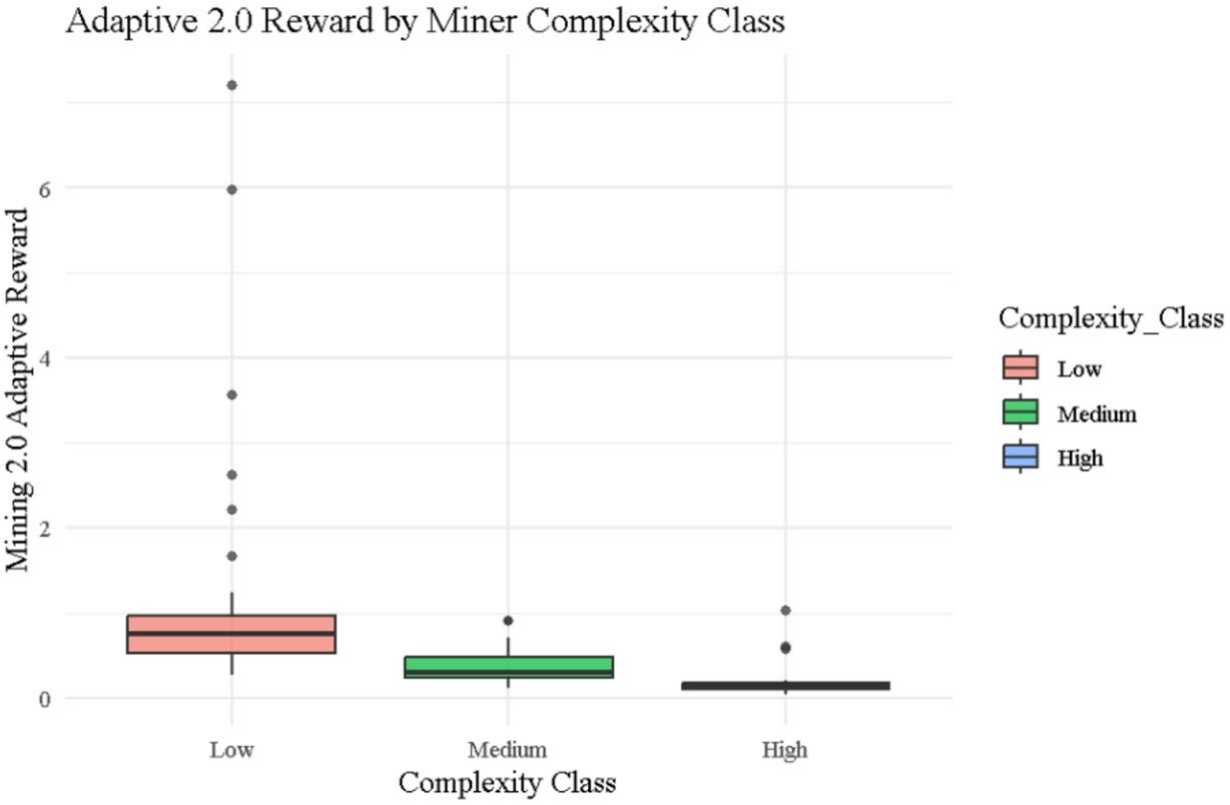
Box plots for Adaptive 2.0 rewards by complexity class.

Figure 9 presents boxplots of rewards distributed under the Adaptive 2.0 protocol across the three miner complexity classes: Low, Medium, and High, based on a reward function that penalizes both $\:{\left.T\right|}_{j}$ and $\:{\left.N\right|}_{j}$. Low complexity miners (red) receive consistently higher and more stable rewards, with a median near 1.0 and notable outliers such as Miner ID 25 achieving a reward of 7.19, and ID 55 earning 2.62, both benefiting from low noise and fast execution as shown in Table 2. Medium complexity miners (green) show lower medians with a compressed reward range typically below 0.6, exemplified by miners like ID 10 and ID 12, whose rewards fall below 0.24. High complexity miners (black) exhibit the most restrictive distribution, with rewards rarely exceeding 0.5 illustrated by ID 23 (0.06), ID 44 (0.05), and ID 13 (0.09) indicating strong suppression due to compounded penalties from noise and complexity. This stratification aligns with the entropy metrics in Table 1, where Adaptive 2.0 achieves the lowest values (Shannon = 1.678, Renyi = 1.396, Tsallis = 0.620, Normalized = 0.723), validating its effectiveness in delivering a fairer, behavior-sensitive reward system that promotes both efficiency and stability.

### Mining 2.0 reward

**Fig. 10:**
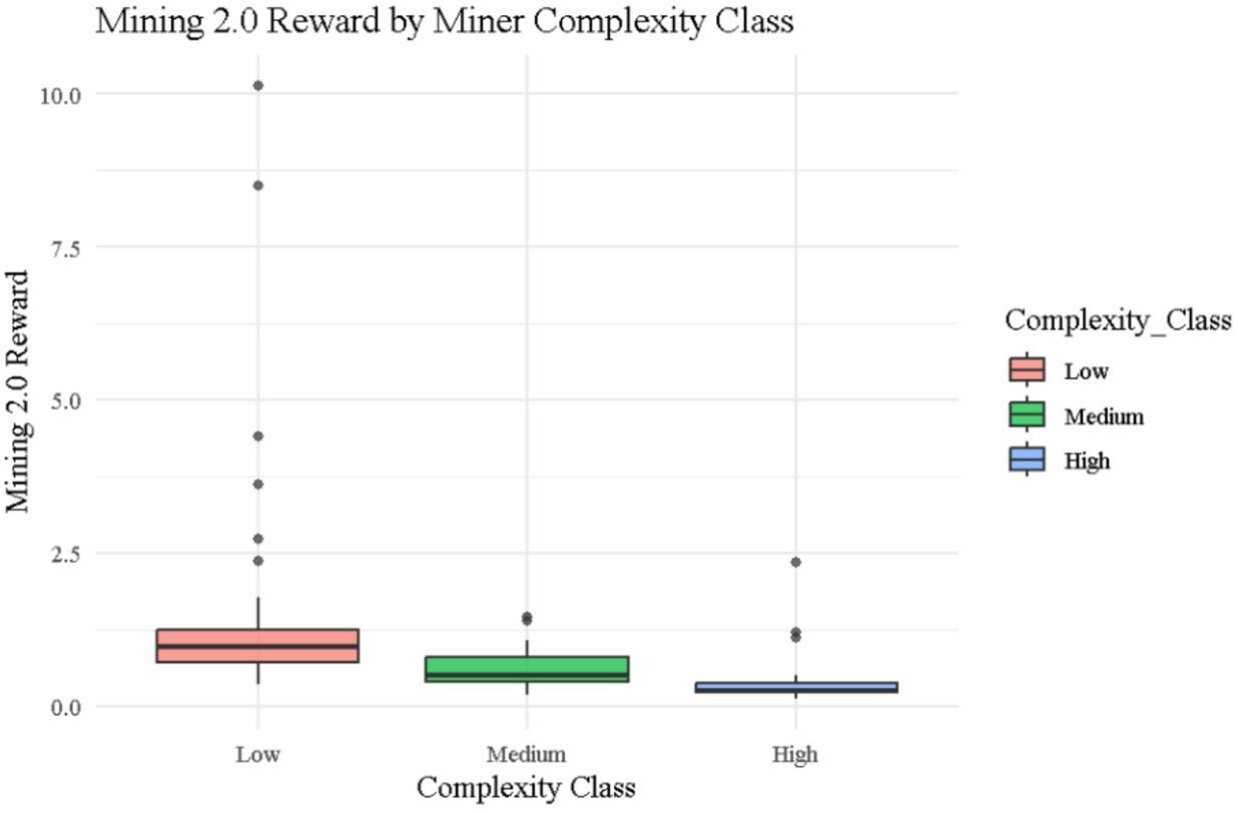
Box plots for mining 2.0 Reward by miner complexity class.

Figure 10 presents the distribution of rewards under the Mining 2.0 protocol across miner complexity classes, reflecting the behavior of the reward function $\:{\left.R\right|}_{j}^{\left(2.0\right)}=\frac{1}{{\left.C\right|}_{j}\times\:{\left.N\right|}_{j}}$​, where $\:{\left.C\right|}_{j}$​ is complexity and $\:{\left.N\right|}_{j}$​ is miner noise. The boxplot highlights that Low complexity miners consistently receive the highest rewards, with several outliers (e.g., ID 25: 10.13, ID 89: 8.50) due to their minimal complexity and exceptionally low noise (0.09872 and 0.11761, respectively). Medium complexity miners show tighter distributions with moderate values (e.g., ID 8: 1.45, ID 22: 1.39), while High complexity miners receive the lowest and most compressed rewards (e.g., ID 23: 0.11, ID 44: 0.13), resulting from compounding penalties from both high $\:{\left.C\right|}_{j}$​ and elevated or unstable noise. This stratified reward behavior aligns precisely with the entropy trends in Table 1, where Mining 2.0 demonstrates reduced disorder (Shannon = 1.885, Tsallis = 0.650, Normalized = 0.729) compared to the Traditional protocol (Shannon = 2.684). Furthermore, Table 2 quantifies this gradient with actual reward values and supports that Mining 2.0 allocates rewards more equitably, targeting behavioral quality and computational efficiency. Together, the visual and numerical evidence confirm Mining 2.0’s role in minimizing randomness, reducing inequality, and promoting protocol-level stability.

### Traditional reward

**Fig. 11:**
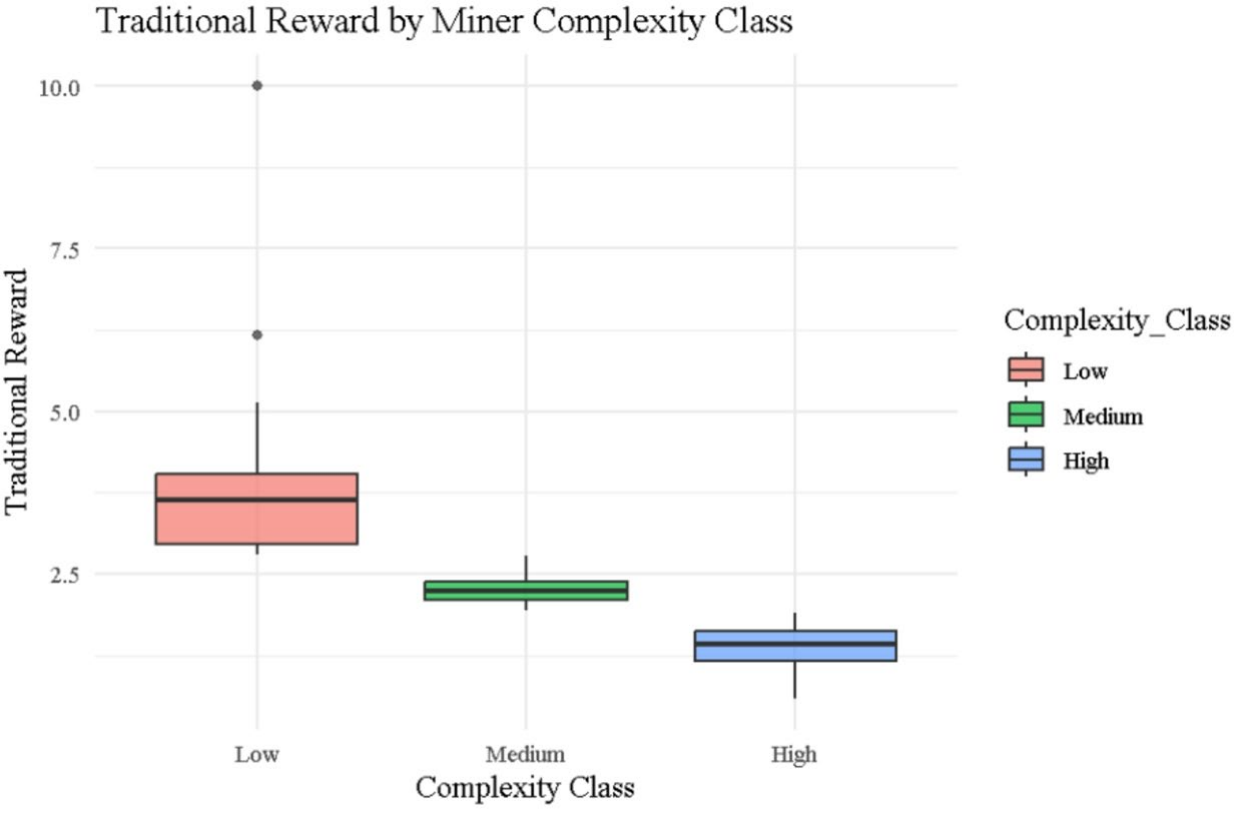
Box plots for traditional rewards by complexity class.

Figure 11 displays boxplots of Traditional rewards segmented by miner complexity class (Low, Medium, High), clearly illustrating a hierarchical drop in reward magnitude as complexity increases. Low complexity miners (red) receive the highest rewards on average, with substantial variability and upper outliers such as Miner ID 9 and ID 25 earning the maximum reward of 10.0 due to extremely low execution times (e.g., $\:{\left.T\right|}_{j}$=1.003and $\:{\left.T\right|}_{j}$=3.416, respectively, from Table 2). Medium complexity miners (green) form a narrower distribution centered around 2.3, while High complexity miners (blue) cluster further down near 1.6, with limited variability—examples include ID 44 and ID 54, who receive 1.11682 and 0.88852, respectively. These results align with the Traditional reward function $\:{\left.R\right|}_{j}^{Traditional}=\frac{1}{{\left.T\right|}_{j}}\times\:\frac{10}{{\left.\mathrm{M}\mathrm{a}\mathrm{x}\right|}_{T}}$​, which excludes miner noise ($\:{\left.N\right|}_{j}$​) and rewards speed alone.

The plot reinforces the design flaw that the Traditional model fails to penalize instability, as seen in Table 2, where miners with high noise levels (e.g., ID 44: $\:{\left.N\right|}_{j}$=2.469, ID 54: $\:{\left.N\right|}_{j}$=1.421) still secure moderate rewards solely due to shorter execution times. This one-dimensional reward logic results in high entropy values in Table 1 (Shannon = 2.684, Normalized = 0.847), reflecting greater randomness and inequity in distribution. Overall, this figure confirms that while Traditional rewards favor speed, they do so at the cost of fairness and behavioral accountability, leading to unstable and inefficient incentive dynamics in the system.

### Shiny web-tool

**Fig. 12:**
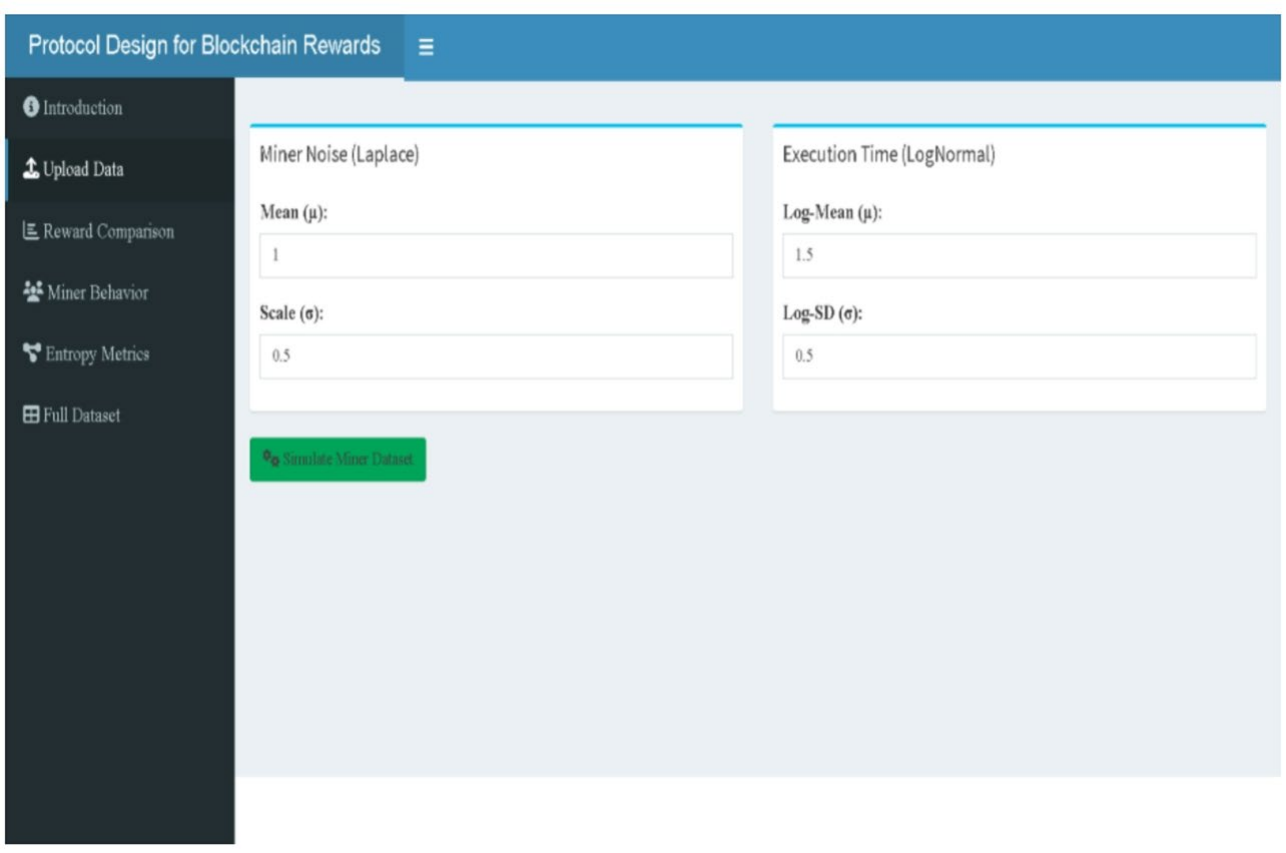
Simulated data generation (web-tool output).

**Fig. 13:**
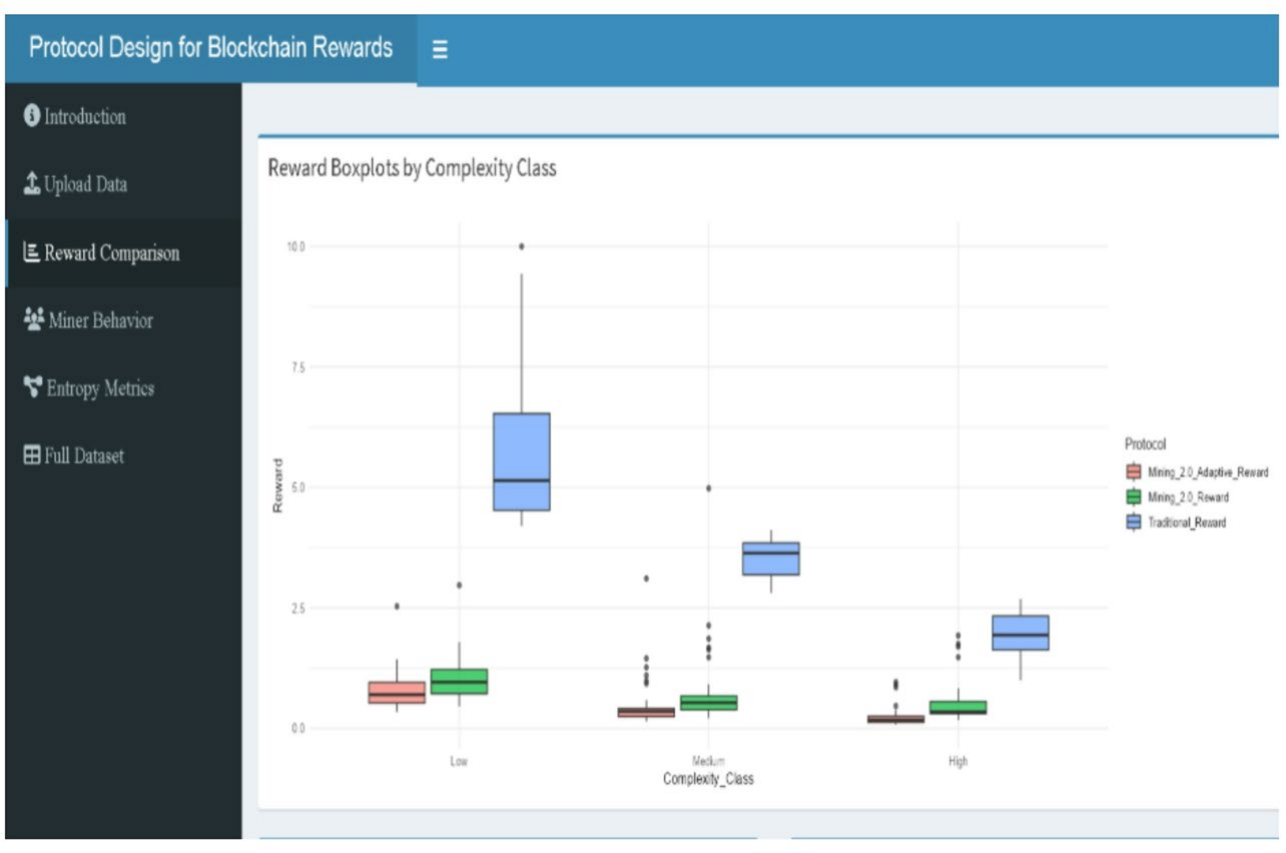
Reward boxplots (web-tool output).

**Fig. 14:**
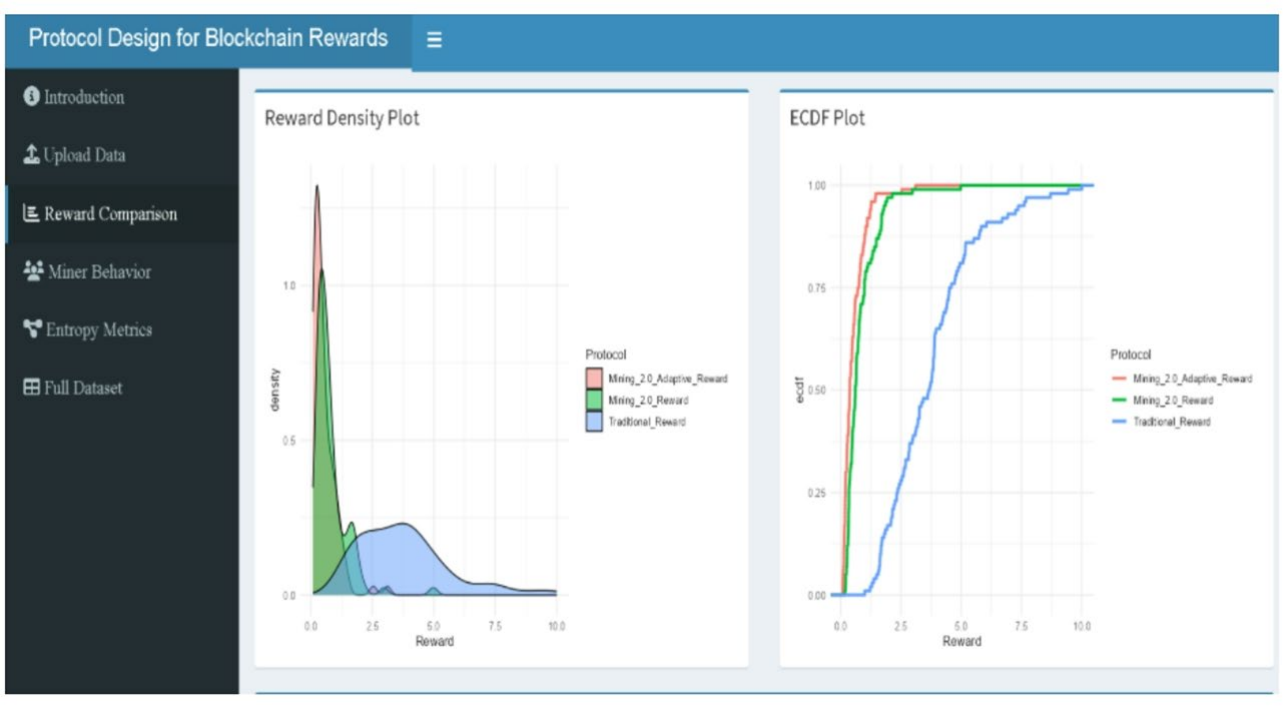
Reward density and ECDF plots (web-tool output).

**Fig. 15:**
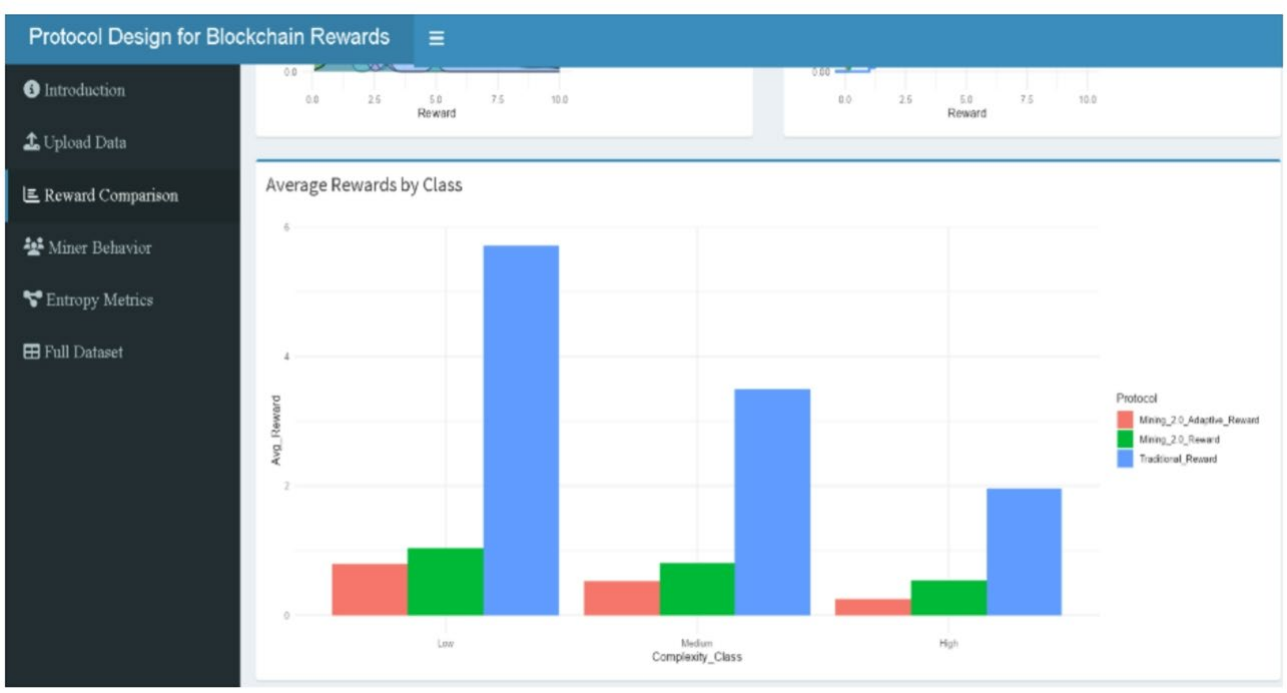
Average Reward (web-tool output).

**Fig. 16:**
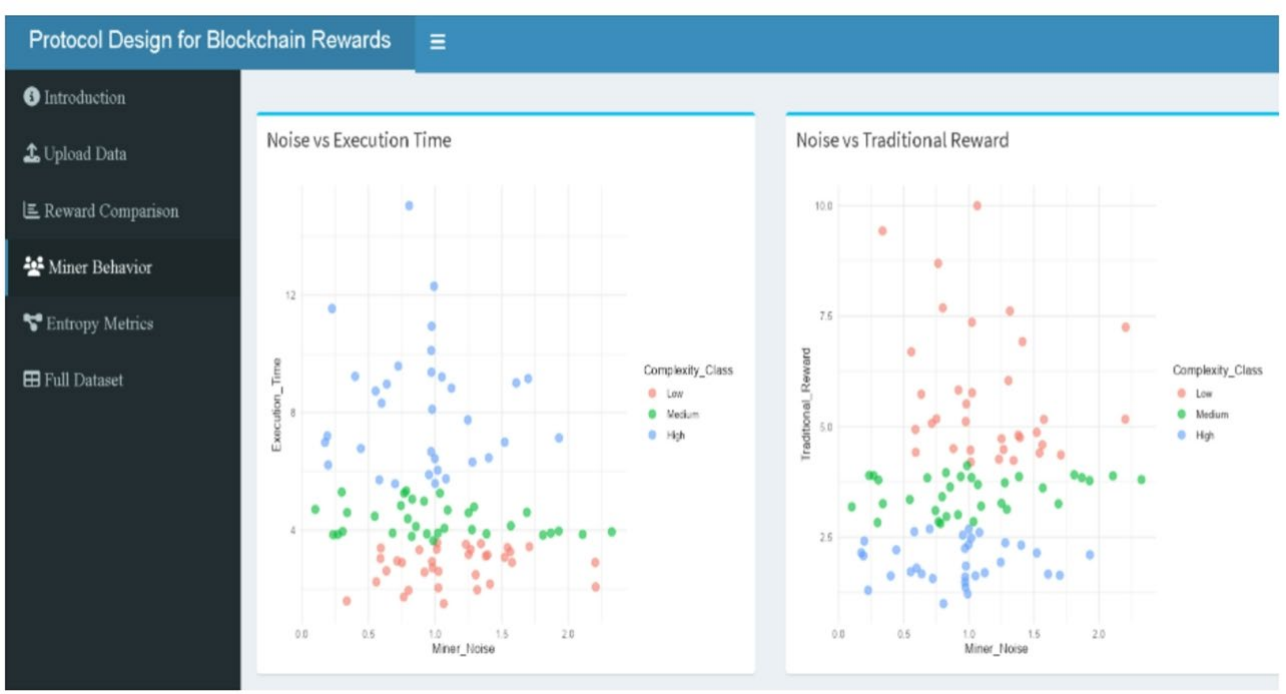
Miner noise vs. execution time and noise vs. Traditional Reward (web-tool output).

**Fig. 17:**
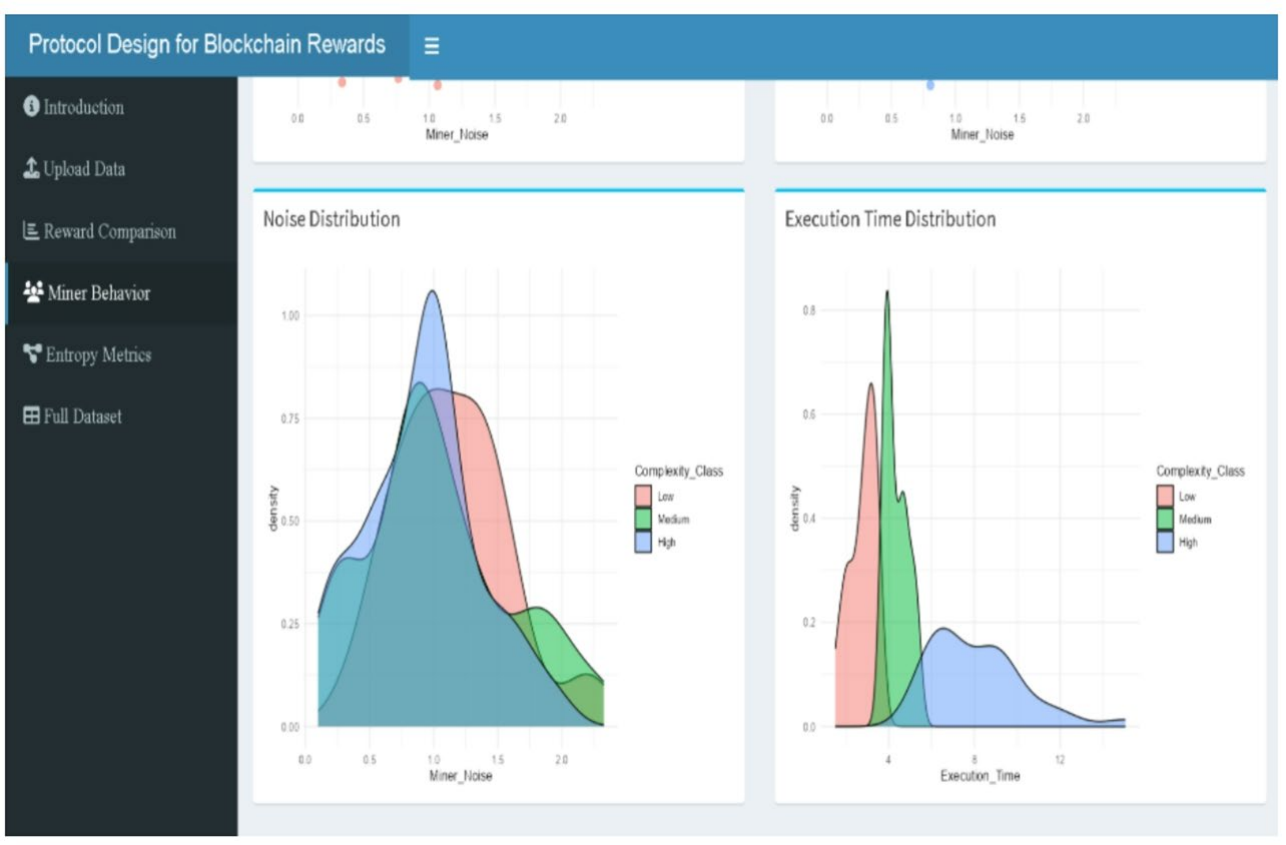
Noise and execution time distributions (web-tool output).

**Fig. 18:**
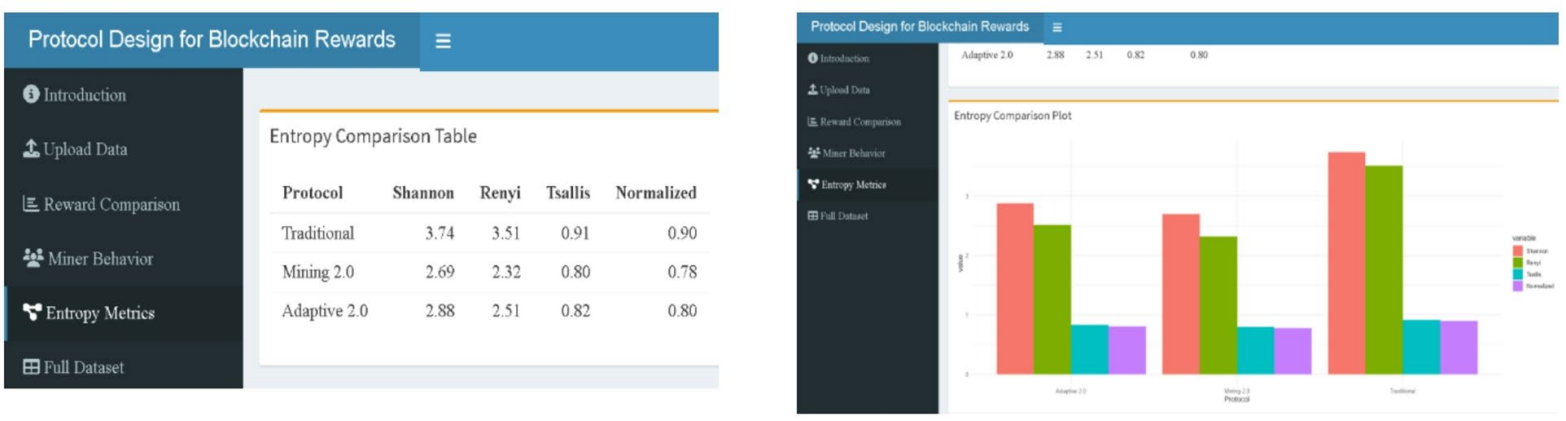
Entropy (left panel) and bar chart (right panel) (web-tool output).

**Fig. 19:**
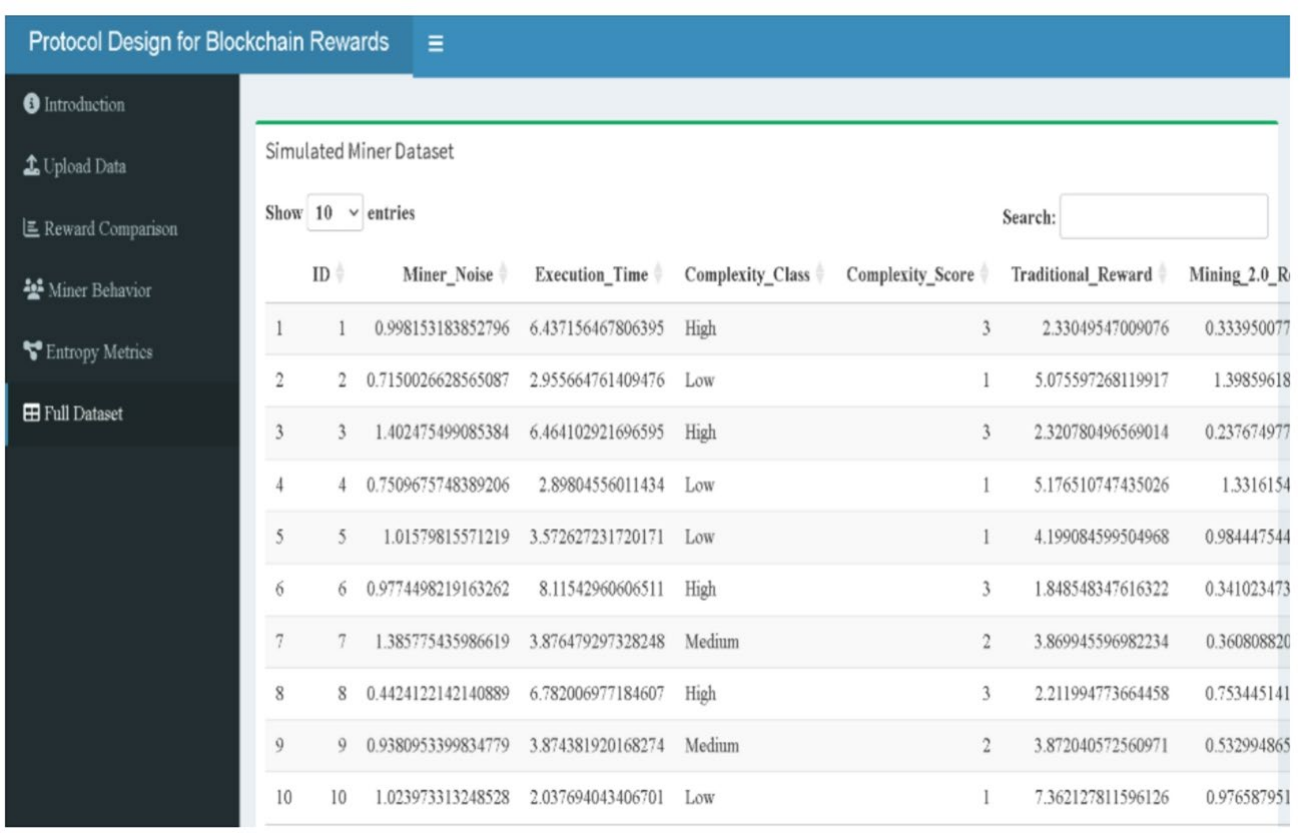
Interactive table for simulated miner dataset (web-tool output).

A Shiny-based web-tool has been developed to provide an intuitive and interactive platform for analyzing blockchain reward mechanisms. Designed for users without programming expertise, the tool facilitates the specification of miner parameters via the Upload Data panel (Fig. 12) and the exploration of three reward structures—Traditional, Mining 2.0, and Adaptive 2.0—based on miner-specific attributes such as execution time and signal instability. The interface is organized into dedicated panels, including reward comparison (boxplots in Fig. 13, density & ECDF in Fig. 14, average rewards in Fig. 15), miner behavior diagnostics (noise vs. time & reward scatterplots in Fig. 16, density distributions in Fig. 17), entropy evaluations (bar chart & table in Fig. 18), and full dataset review (Fig. 19).

Built using R, the application is hosted on the free R Shiny server (www.shinyapps.io), ensuring open and cost-free access. It leverages widely used R packages—shiny, shinydashboard, ggplot2, dplyr, tidyr, patchwork, entropy, reshape2, and DT—to offer a seamless and versatile user experience. Minor differences in generated plots and tables may occur due to the stochastic nature of the simulated data in each session; however, these variations do not alter the overall interpretability or consistency of the findings presented through the tool.

### Sensitivity and ablation analysis

**Fig. 20:**
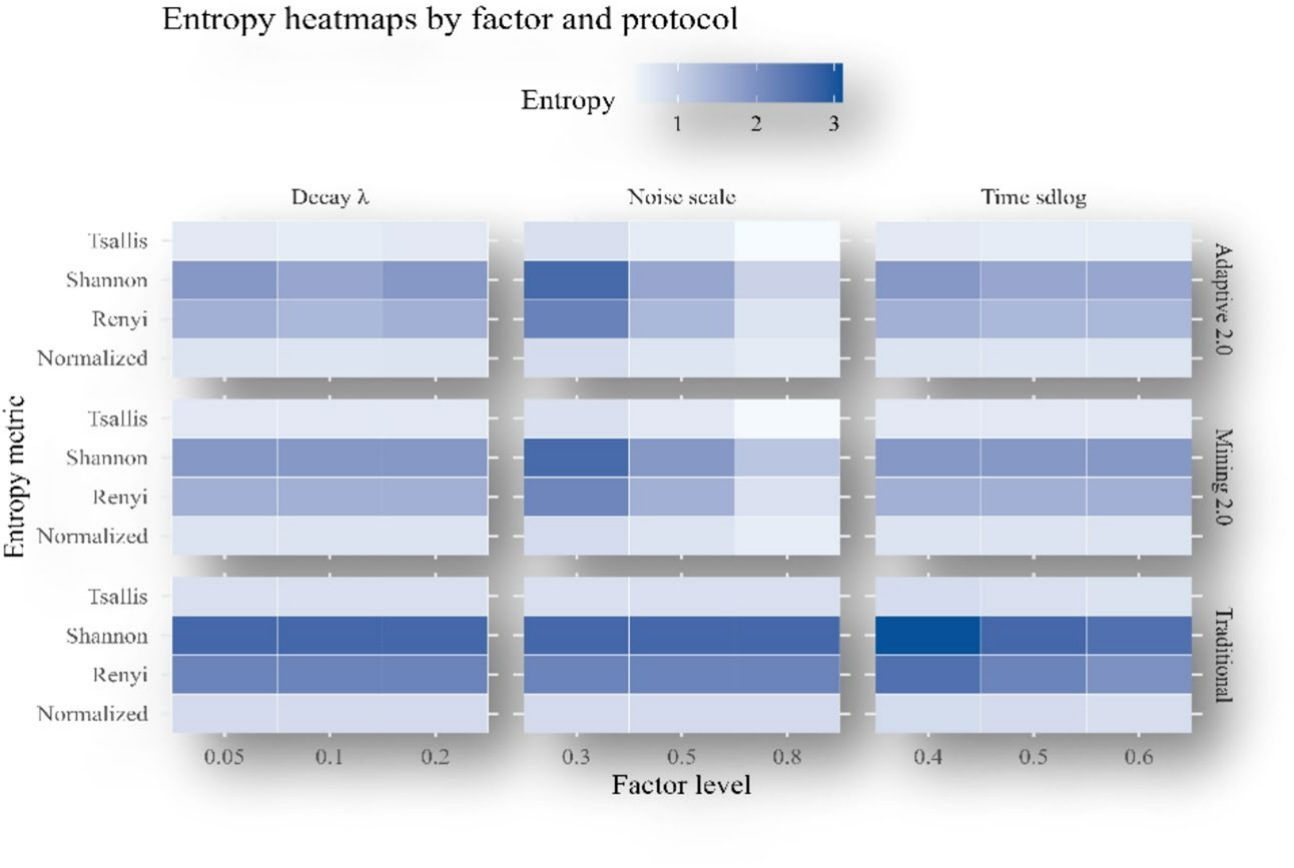
Entropy Heatmap by Factor and Protocol.

**Fig. 21:**
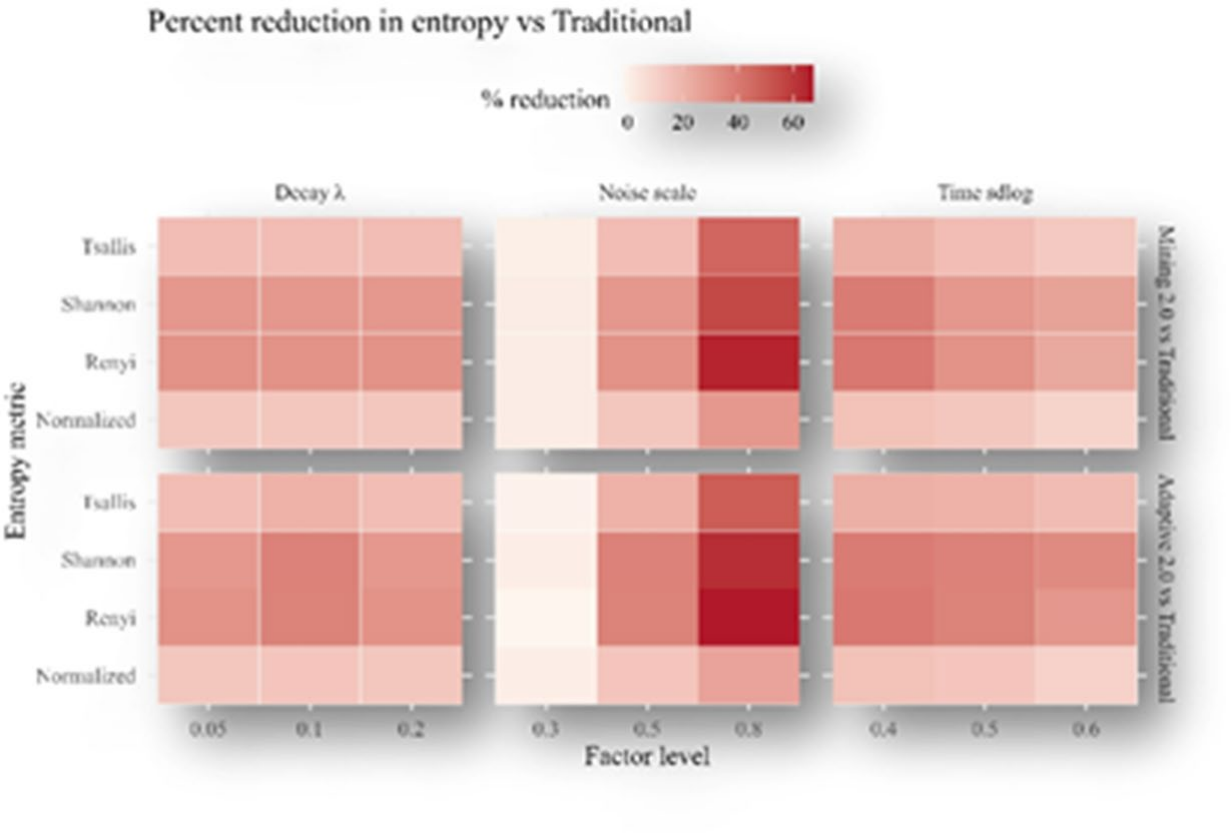
Entropy reduction Heatmap vs. Traditional.

We conducted one-factor-at-a-time sweeps around the baseline ($\:n=100;\:20$-bin discretization; fixed seed): Laplace noise scale $\:b\in\:(\mathrm{0.3,0.5,0.8}$), execution-time sdlog $\:\in\:(\mathrm{0.4,0.5,0.6}$), and adaptive decay $\:{\uplambda\:}\in\:(\mathrm{0.05,0.10,0.20}$). For each setting, rewards were recomputed under all three protocols and summarized by Shannon, Rényi-2, Tsallis-2, and normalized entropies. Results are visualized as heatmaps of $\:\left(i\right)$ absolute entropies (Fig. 20) and $\:\left(ii\right)$ percent reductions of Mining 2.0 and Adaptive 2.0 relative to Traditional (Fig. 21).

- As noise scale (see $\:b$) increases, entropy rises for Mining 2.0 and Adaptive 2.0 across all metrics, reflecting greater allocation disorder from instability; Traditional which does not depend on noise—remains essentially unchanged. Consequently, the relative improvement over Traditional strengthens with higher noise scale ($\:see\:b$) (Fig. 20), with Adaptive 2.0 consistently dominating Mining 2.0.
- Larger sdlog yields higher entropy for all protocols and metrics (Fig. 20), indicating more unequal allocations when runtimes are more dispersed. Despite this, the protocol ordering is preserved: Adaptive 2.0 $\:<$ Mining 2.0 $\:<$ Traditional. Percent-reduction heatmaps (Fig. 21) show moderate increases in gains for the behavior-aware protocols as dispersion grows.
- Increasing ($\:{\uplambda\:}$) reduces entropy for Adaptive 2.0 (lighter tiles in Fig. 20), while Mining 2.0 and Traditional are largely unaffected, as expected. The percentage-reduction panels (Fig. 21) show a corresponding enhancement of Adaptive 2.0’s advantage.

Across all factors–level–metric combinations examined, the ranking remains stable Adaptive 2.0 achieves the lowest entropy, Mining 2.0 is intermediate, and Traditional is highest demonstrating the robustness of the main findings to noise intensity, runtime dispersion, and decay strength.

## Discussion

This study compared three reward mechanisms Traditional, Mining 2.0, and Adaptive 2.0 under a controlled but heterogeneous miner population. The Traditional rule, which depends only on execution time, generated the broadest and most right-tailed reward distribution: on the normalized scale, rewards span roughly 0.58–10.00 and concentrate disproportionately among the fastest miners, even when their behavior is noisy. Because instability and complexity are ignored, slower yet stable miners are systematically penalized, a pattern that is visible in the density/ECDF displays and confirmed by miner-level summaries (Table 2). Introducing behavior awareness in Mining 2.0 compresses the distribution by down-weighting ($\:1/\left(\mathrm{noise}\times\:\mathrm{complexity}\right)$); the support remains wide ($\:\approx\:0.11-10.13$) but the mass shifts toward moderate rewards and away from unstable or high-complexity outliers. Adaptive 2.0 adds an exponential time penalty and yields the tightest allocation ($\:\approx\:0.05-7.20$), effectively curbing long-runtime tails while preserving sensitivity to stability and task difficulty. Entropy metrics make these contrasts quantitative: relative to Traditional, Mining $\:2.0$ lowers Shannon, Rényi-$\:2$, Tsallis-$\:2$ and normalized entropies by $\:29.8\%,\:31.7\%,\:17.2\%,$ and $\:13.9\%,$ respectively, while Adaptive $\:2.0$ achieves $\:37.5\%,\:37.1\%,\:21.0\%,$ and $\:14.6\%$ reductions (Table 1). The ordering Adaptive $\:2.0\:<$ Mining $\:2.0\:<$ Traditional is consistent across all four metrics and all plots.

To assess robustness, we conducted one-factor-at-a-time sweeps around the baseline: the Laplace noise scale ($\:b\in\:\mathrm{0.3,0.5,0.8}$), the execution-time dispersion ($\:\mathrm{sdlog}\in\:\mathrm{0.4,0.5,0.6}$), and the adaptive decay ($\:{\uplambda\:}\in\:\mathrm{0.05,0.10,0.20}$). Heatmaps of absolute entropy (Fig. 20) and of percent reductions versus Traditional (Fig. 21) show clear and interpretable trends. Increasing noise scales monotonically raises entropy for the behavior-aware protocols across all metrics, reflecting the added disorder from instability, yet the relative advantage over Traditional strengthens as noise scales grows tiles darken in Fig. 20 for both Mining 2.0 and Adaptive 2.0 with Adaptive 2.0 dominant throughout. Greater runtime dispersion (larger $\:\left(\mathrm{sdlog}\right)$) similarly elevates entropy for every protocol and metric, but the ranking remains unchanged, indicating that behavior-aware penalization continues to improve equity even when execution times are broadly spread. Varying ($\:{\uplambda\:}$) has the expected selective effect: higher ($\:{\uplambda\:}$) lowers entropy for Adaptive 2.0 and leaves Mining 2.0 and Traditional essentially unchanged, confirming that the decay term is the primary driver of Adaptive 2.0’s additional stability. Across all factors–level–metric combinations examined, the protocol ordering by entropy never reverses, demonstrating that the main fairness conclusions are not artefacts of a narrow parameter choice.

These results have direct design implications. Mining 2.0 is a simple modification that provides sizeable stability gains with minimal modelling overhead, suitable when implementers want a low-complexity to change that still discourages noisy or high-complexity behavior. Adaptive 2.0 delivers the largest and most robust reductions in disorder by explicitly penalizing prolonged runtimes; in practice, monitoring entropy alongside throughput and latency would allow operators to tune ($\:{\uplambda\:}$) to maintain low-entropy reward distributions as network conditions evolve.

The analysis is necessarily bound by its simulation setup. Execution times and noise were generated from log-normal and Laplace laws and discretized into $\:20$ bins for entropy estimation; alternative data-generating processes or binning schemes could shift absolute values, although the observed ordering and trends proved stable under the sensitivity sweeps. Entropy measures capture distributional uncertainty and equality but not economic utility or strategic adaptation over long horizons. Moreover, we treat complexity and instability as independent factors; in operational systems they may be correlated, which could sharpen or dampen the measured effects. To bridge this gap, the accompanying Shiny application enables parameter exploration and can be extended to ingest telemetry from test-nets or public clients, allowing calibration against measured traces and end-to-end validation of reward rules. Within these bounds, the evidence indicates a clear progression: the baseline rewards speed alone and exhibits the highest disorder; Mining 2.0 aligns payouts with behavioral quality; and Adaptive 2.0 most effectively suppresses disorder while preserving responsiveness to miner heterogeneity.

The present evidence is derived from controlled simulations $\:\left(T,\eta\:,s\right)\sim\:\left(\mathrm{LogNormal},\mathrm{Laplace},\mathrm{terciles}\right)$ with fixed discretization ($\:m=20$), so our results pertain to a model distribution $\:p\left(T,\eta\:,s\right)\ne\:{p}^{\mathrm{*}}\left(T,\eta\:,s\right)$ in practice. To establish external validity, the next phase is a deployment study on mature stacks $\:G$ = {Bitcoin Core regtest, geth dev chains, Hyperledger/Tendermint}. For each protocol $\:\left(k\in\:\mathrm{Trad},\mathrm{M2},\mathrm{Ad}\right)$, we will implement the payout rule (R_k) and evaluate the metric vector$\:Mk={H}_{S},{H}_{R2},{H}_{T2},{H}_{\mathrm{norm}},\:\tau\:\left(\mathrm{throughput}\right),\mathcal{l}\left(\mathrm{latency}\right),\mathfrak{o}\left(\mathrm{orphan}\mathrm{/}\mathrm{fork}\right),\mathcal{e}\left(\mathrm{energy}\right)$.

on block-level samples. Our primary hypotheses are:$\:{H}_{1}:\:{H}^{Adaptive}<{H}^{\mathrm{M2}}<{H}^{Traditional}\:\:\mathrm{(rank\:preserved)},\hspace{1em}$

$$\:{H}_{2}:\:\varDelta\:{H}^{Adaptive}={H}^{Traditional}-{H}^{Adaptive}>0,\:$$

$$\:\varDelta\:{H}^{\mathrm{M2}}>0,$$

with non-regression constraints $\:\left({\tau\:}_{k}\approx\:{\tau\:}_{\mathrm{Trad}},\:{\mathcal{l}}_{k}\approx\:{\mathcal{l}}_{\mathrm{Trad}}\right)$within tolerance $\:{\upepsilon\:}$. We will report bootstrap CIs for $\:\left({\Delta\:}H\right)$, and Kendall’s $\:\left({{\uptau\:}}_{b}\right)$ for rank agreement, using replayed traces and controlled loads; all scripts and logs will be released. This plan turns our framework from a controlled mechanism study into a testable, system-level validation.

## Conclusions and future study

This work presented a structured comparison of three blockchain reward mechanisms Traditional (following Nakamoto^11^, Mining $\:2.0$, and Adaptive $\:2.0$ under heterogeneous miner behavior. Using distributional diagnostics (KDE/ECDF) and four entropy measures on discretized rewards, we showed that behavior-aware designs reduce allocation disorder and improve equity. Mining $\:2.0$, which penalizes instability and task complexity, consistently outperforms the execution-time-only baseline; Adaptive $\:2.0$, which additionally penalizes prolonged runtimes, achieves the lowest entropy across metrics. An accompanying Shiny application operationalizes the framework, enabling transparent, reproducible exploration without programming.

Several challenges temper these findings. Results are derived from controlled simulations with specific parametric choices for execution time and noise, tercile complexity classes, and fixed discretization; these facilitate isolation of mechanism effects but do not fully reflect operational variability, strategic behavior, or potential correlation between complexity and instability.

Entropy provides a rigorous lens on distributional uncertainty and fairness, yet it does not directly capture miner utility, welfare trade-offs with throughput/latency/energy, or governance and implementation overheads. Sensitivity analyses indicate that qualitative rankings are robust to noise intensity, runtime dispersion, and decay strength, but absolute levels may shift under alternative workloads and network conditions.

Future study will move from controlled mechanism analysis to system-level validation and theory-guided design. We will implement the payout rules on mature open-source stacks (e.g., Bitcoin Core regtest, geth dev chains, Hyperledger/Tendermint) and evaluate a joint metric set spanning entropies and operational KPIs (throughput, latency, orphan/fork rate, energy). We will explore principled parameterization deriving decay and penalty schedules from incentive-compatibility or information-theoretic objectives and test robustness under adversarial, collusive, and hardware-heterogeneous regimes. Extensions include integrating additional equity metrics (e.g., Gini/Theil), adaptive parameter tuning via online learning, generalization to non-PoW settings, and augmenting the Shiny tool to ingest empirical traces for calibration and large-scale scenario testing.

## Data Availability

All data supporting the findings of this study are reproducibly generated through the authors’ Shiny web-tool, which enables dynamic simulation of miner behavior and reward protocols; no external datasets were used, ensuring full transparency and replicability.

## Appendix

See Table 2.

**Table 2:** Miner-level metrics and reward outcomes across protocols.

Miner ID	Complexity Score	Complexity Class	Miner Noise	Execution Time	Traditional Reward	Mining 2.0 Reward	Mining 2.0 Adaptive
1	3	High	1.88484	5.26438	1.90613	0.17685	0.10447
2	1	Low	2.03634	3.02854	3.31335	0.49108	0.36276
3	3	High	0.72094	9.85386	1.01834	0.46236	0.17260
4	3	High	1.54072	6.18081	1.62351	0.21635	0.11661
5	2	Medium	1.16668	4.68741	2.14076	0.42857	0.26819
6	2	Medium	1.01947	5.14629	1.94987	0.49045	0.29315
7	3	High	1.32044	6.29430	1.59424	0.25244	0.13452
8	2	Medium	0.34410	4.68758	2.14068	1.45308	0.90931
9	1	Low	1.18843	1.00346	10.0000	0.84145	0.76111
10	2	Medium	1.26393	5.16777	1.94177	0.39559	0.23595
11	2	Medium	0.95585	3.72990	2.69031	0.52310	0.36024
12	2	Medium	1.28833	4.91659	2.04097	0.38810	0.23737
13	3	High	2.01760	5.99492	1.67385	0.16521	0.09072
14	3	High	0.66417	9.02383	1.11201	0.50188	0.20356
15	1	Low	0.96080	3.11539	3.22098	1.04080	0.76220
16	3	High	2.06025	8.59578	1.16739	0.16179	0.06849
17	3	High	2.56696	5.30115	1.89291	0.12986	0.07642
18	3	High	0.27586	7.53270	1.33214	1.20834	0.56891
19	3	High	0.97435	7.10191	1.41294	0.34211	0.16816
20	3	High	1.06429	6.42656	1.56143	0.31320	0.16471
21	1	Low	1.82529	2.66030	3.77198	0.54786	0.41989
22	2	Medium	0.35889	4.28408	2.34230	1.39319	0.90772
23	3	High	2.90346	6.12121	1.63932	0.11481	0.06225
24	1	Low	2.11904	2.78219	3.60673	0.47191	0.35730
25	1	Low	0.09872	3.41639	2.93719	10.13002	7.19843
26	3	High	1.01442	5.99244	1.67455	0.32860	0.18047
27	3	High	0.87603	6.58036	1.52493	0.38050	0.19705
28	3	High	1.83427	5.65129	1.77563	0.18173	0.10327
29	1	Low	0.94394	2.87805	3.48660	1.05939	0.79444
30	1	Low	1.55738	2.58599	3.88037	0.64210	0.49579
31	3	High	1.32236	9.54821	1.05094	0.25207	0.09702
32	2	Medium	1.48658	5.09857	1.96812	0.33634	0.20200
33	2	Medium	0.87334	4.68432	2.14217	0.57252	0.35839
34	2	Medium	1.23129	4.21880	2.37854	0.40608	0.26631
35	1	Low	1.42066	2.46659	4.06822	0.70390	0.55003
36	3	High	1.54806	6.08605	1.64879	0.21532	0.11716
37	2	Medium	1.11103	4.02060	2.49580	0.45003	0.30104
38	2	Medium	0.56064	4.09031	2.45326	0.89183	0.59244
39	3	High	1.83887	7.14686	1.40406	0.18127	0.08870
40	3	High	1.12652	6.75908	1.48461	0.29590	0.15052
41	3	High	0.86220	8.98951	1.11626	0.38661	0.15735
42	1	Low	0.93126	3.53217	2.84092	1.07382	0.75428
43	3	High	0.29605	6.20388	1.61747	1.12592	0.60545
44	3	High	2.46949	8.98499	1.11682	0.13498	0.05496
45	1	Low	0.92662	2.57180	3.90179	1.07919	0.83446
46	1	Low	2.23345	2.91422	3.44332	0.44774	0.33455
47	1	Low	1.74696	2.54500	3.94287	0.57242	0.44380
48	1	Low	1.16422	2.16062	4.64433	0.85894	0.69204
49	2	Medium	2.42308	4.66455	2.15125	0.20635	0.12943
50	3	High	1.13569	6.21274	1.61517	0.29351	0.15769
51	3	High	0.79741	8.17011	1.22821	0.41802	0.18466
52	3	High	0.81700	7.55625	1.32799	0.40800	0.19164
53	1	Low	0.88653	2.71392	3.69745	1.12799	0.85989
54	3	High	1.42127	11.29365	0.88852	0.23453	0.07581
55	1	Low	0.27634	3.21110	3.12498	3.61876	2.62484
56	2	Medium	1.34417	4.72448	2.12396	0.37198	0.23192
57	2	Medium	1.21891	3.62869	2.76535	0.41020	0.28537
58	2	Medium	0.46430	4.21574	2.38027	1.07689	0.70645
59	2	Medium	0.67512	4.92388	2.03795	0.74060	0.45263
60	2	Medium	1.01462	4.75683	2.10952	0.49279	0.30625
61	2	Medium	1.21633	4.42581	2.26729	0.41107	0.26406
62	2	Medium	2.68535	4.73053	2.12125	0.18620	0.11602
63	1	Low	1.36604	3.51585	2.85410	0.73205	0.51505
64	1	Low	1.07134	3.48299	2.88103	0.93341	0.65888
65	1	Low	1.60095	1.95316	5.13762	0.62463	0.51380
66	2	Medium	0.51482	3.70185	2.71070	0.97121	0.67072
67	1	Low	0.69428	3.46834	2.89321	1.44033	1.01820
68	3	High	1.53402	17.30414	0.57990	0.21729	0.03851
69	1	Low	1.24421	2.26810	4.42424	0.80372	0.64062
70	2	Medium	0.63415	4.80006	2.09052	0.78846	0.48788
71	1	Low	0.22683	2.12376	4.72493	4.40850	3.56498
72	1	Low	0.36523	2.14853	4.67046	2.73804	2.20866
73	2	Medium	0.58123	4.77002	2.10368	0.86025	0.53391
74	1	Low	0.97896	2.72285	3.68533	1.02149	0.77800
75	2	Medium	0.53534	4.47761	2.24107	0.93399	0.59687
76	2	Medium	1.28876	3.61782	2.77366	0.38797	0.27020
77	1	Low	1.07484	3.29750	3.04310	0.93037	0.66903
78	1	Low	0.85681	1.62850	6.16186	1.16712	0.99172
79	1	Low	1.01462	2.42936	4.13056	0.98559	0.77302
80	2	Medium	1.88159	4.90256	2.04681	0.26573	0.16275
81	3	High	1.08909	5.95249	1.68578	0.30607	0.16877
82	1	Low	0.42369	3.50280	2.86474	2.36020	1.66274
83	2	Medium	0.83440	4.48183	2.23895	0.59924	0.38278
84	3	High	1.17214	7.85731	1.27710	0.28438	0.12962
85	3	High	1.40109	9.20667	1.08993	0.23791	0.09475
86	1	Low	1.06808	2.58944	3.87520	0.93626	0.72267
87	2	Medium	0.61972	4.22636	2.37429	0.80681	0.52872
88	3	High	0.14249	8.17229	1.22788	2.33930	1.03316
89	1	Low	0.11761	3.54357	2.83178	8.50275	5.96573
90	2	Medium	0.75321	4.36564	2.29854	0.66383	0.42900
91	2	Medium	1.20387	4.29283	2.33753	0.41533	0.27037
92	1	Low	2.82317	2.87531	3.48992	0.35421	0.26570
93	1	Low	0.56283	3.58823	2.79654	1.77673	1.24104
94	2	Medium	2.00521	4.41619	2.27223	0.24935	0.16033
95	2	Medium	1.95288	3.64394	2.75378	0.25603	0.17784
96	3	High	1.31573	7.82007	1.28319	0.25334	0.11590
97	1	Low	0.79688	3.52367	2.84777	1.25490	0.88222
98	2	Medium	1.01529	3.60894	2.78048	0.49247	0.34328
99	3	High	1.33467	6.34985	1.58029	0.24975	0.13235
100	1	Low	1.13611	2.64274	3.79705	0.88019	0.67578
